# Plasmid Crosstalk in Cell-Free Expression Systems

**DOI:** 10.1021/acssynbio.3c00412

**Published:** 2023-09-27

**Authors:** Fernanda Piorino, Alexandra T. Patterson, Yue Han, Mark P. Styczynski

**Affiliations:** † School of Chemical and Biomolecular Engineering, Georgia Institute of Technology, 311 Ferst Drive NW, Atlanta, Georgia 30332-0100, United States

**Keywords:** Cell-free systems, plasmid crosstalk, genetic circuits, resource competition, ribonuclease distraction, toxic metabolite buildup

## Abstract

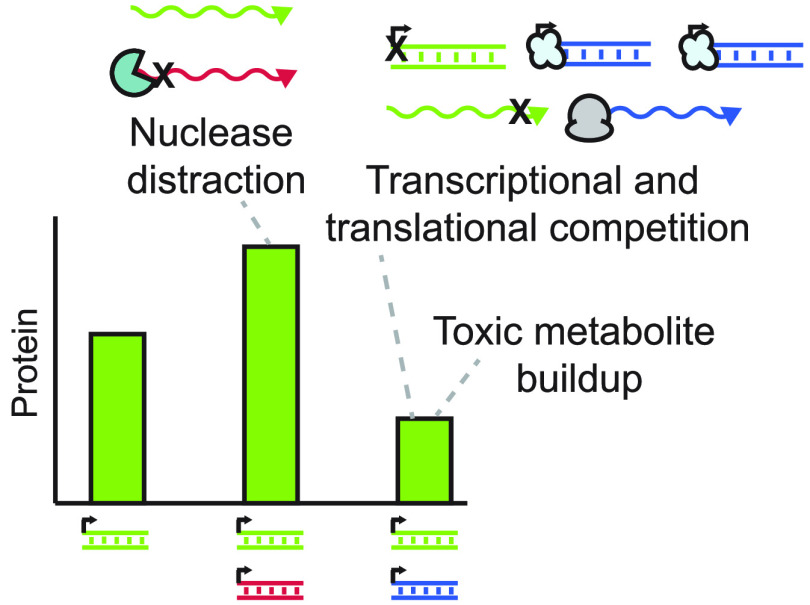

Although cell-free protein expression has been widely used for the synthesis of single proteins, cell-free synthetic biology has rapidly expanded to new, more complex applications. One such application is the prototyping or implementation of complex genetic networks involving the expression of multiple proteins at precise ratios, often from different plasmids. However, expression of multiple proteins from multiple plasmids may inadvertently result in unexpected, off-target changes to the levels of the proteins being expressed, a phenomenon termed plasmid crosstalk. Here, we show that the effects of plasmid crosstalk—even at the qualitative level of increases vs decreases in protein expression—depend on the concentration of plasmids in the reaction and the type of transcriptional machinery involved in the expression. This crosstalk can have a significant impact on genetic circuitry function and even interpretation of simple experimental results and thus should be taken into consideration during the development of cell-free applications.

## Introduction

Cell-free expression (CFE) systems have become a powerful synthetic biology tool.^1,2^ Based on purified cellular machinery (e.g., PURE systems)^3^ or crude extracts, CFE systems enable *in vitro* transcription and translation for production of proteins. Since these systems are not living, they are not subject to biochemical constraints associated with cell survival and growth. This in turn means that they have the potential to enable more efficient allocation of biochemical resources to production of proteins or metabolites and to enable the production of molecules that would otherwise be too toxic to produce in whole cells. In addition, the membraneless and open nature of these systems allows direct control over reaction components, making it simple to test, tune, and tailor the reaction conditions for a given application.

CFE systems have been most commonly envisioned for use in biomanufacturing to enable high-yield expression of a protein.^4,5^ In these applications, a single plasmid encoding the target gene under control of a strong promoter would be added to a cell-free reaction to produce high protein titers. Commonly used for this purpose is a promoter taken from the T7 phage, which when combined with T7 RNA polymerase (the typical polymerase in PURE systems and widely available in lysate-based systems) yields stronger expression than native bacterial systems.^6^

More complex applications may involve the simultaneous expression of multiple proteins to implement genetic circuits, sometimes requiring the use of endogenous bacterial promoters and transcriptional machinery. Since PURE systems do not typically contain bacterial polymerases, they are frequently not suitable for such applications;^3,7^ lysate-based cell-free expression has been particularly useful in these cases. However, the shift from purified recombinant elements in a mostly defined mixture to a cellular lysate with complex and potentially varying compositions can present unique challenges of its own.

Nonetheless, tuning complex genetic circuits in lysate-based cell-free systems is much more straightforward compared to whole-cell *in vivo* implementations. In whole-cell systems, gene expression is typically tuned by altering promoter and ribosomal binding site (RBS) strengths, a process that requires substantial effort and time. In CFE systems, gene expression is typically controlled much more simply by changing the amount of plasmid added to the cell-free reaction. In multiprotein networks, each protein can be expressed from a separate plasmid at any user-defined concentration.

However, expressing multiple genes from potentially multiple plasmids may result in unexpected, off-target changes to protein levels where the addition of a second plasmid to a reaction may affect protein expression from the first plasmid even if the two plasmids do not express proteins that share regulatory interactions.^8^ This “crosstalk” may preclude accurate assessment of function in circuits, potentially masking true regulatory interactions or giving the appearance of regulatory interactions when there are none. This phenomenon has been previously observed and studied for *in vivo* systems, and predictive computational models have been developed.^9,10^ However, these models are likely insufficient for predicting crosstalk in CFE, which are fundamentally different from living cells.^11^ For CFE systems to provide an impactful platform for prototyping complex genetic circuits, an in-depth understanding of crosstalk is needed so that it can be predicted and controlled for.

Crosstalk in CFE systems has already been studied to some extent. It has often been associated with resource competition,^8,12,13^ as there is a limited, shared pool of transcriptional and translational resources in the cell extract—most notably, RNA polymerases and ribosomes—that may define a maximum total expression capacity in a reaction. Several studies have highlighted negative crosstalk arising from resource competition and explored these effects in the context of varying plasmid dosages^8,14^ and *E. coli* promoters of different strengths.^8^ Mathematical models have also been developed to capture these negative effects.^14−17^ One of them^17^ predicted a finite combination of protein concentrations that can be achieved simultaneously in a cell-free reaction, underscoring the “constraint” that one genetic cassette imposes on the other, which has been borne out to some extent experimentally. Resource competition has even been used to implement a cell-free oscillator system.^18^

While significant, competition for transcriptional and translational resources cannot account for all observed crosstalk in CFE systems. While resource competition is expected to lead to a decrease in protein levels, crosstalk has also been experimentally observed to cause off-target increases in protein expression.^8^ It was speculated that the expression of a second gene generates additional mRNA transcripts that “compete” for RNases that are also limited in the reaction, allowing other transcripts to survive longer in the reaction. However, this phenomenon—along with many other details in the mechanisms and impacts of crosstalk—has been insufficiently studied to allow for it to be accounted for in the design of complex cell-free genetic circuits.

Here, we move toward a more thorough investigation and characterization of crosstalk between plasmids in *E. coli*-based CFE systems. We use multiple reporters to decouple the effects of transcription and translation, and we study the effects of plasmid dosage, polymerase type, promoter strength, and even other plasmid components on crosstalk. Despite quantitative variability in effects across lysate batches, we observe qualitatively consistent trends that lead to hypothesized mechanisms for the causes of different types of crosstalk. Finally, we demonstrate two prototypical and realistic examples of how plasmid crosstalk can confound the results of simple experiments, highlighting the importance of accounting for plasmid crosstalk either in models or via careful design of experimental controls.

## Results and Discussion

### Plasmid Crosstalk with “Empty” Vectors

To begin to characterize plasmid crosstalk in CFE systems, we added different “empty” vectors to cell-free reactions expressing protein from different reporter plasmids. To quantify the impact of crosstalk, we used a “crosstalk ratio”: the ratio of fluorescence when a second plasmid is present in the reaction to the fluorescence produced by the reporter plasmid alone, with values greater than and less than 1 denoting positive and negative effects of crosstalk, respectively.

The reporter plasmid expressed superfolder GFP (sfGFP) from a strong RBS under one of four promoters: (1) a strong T7 promoter (P_T7,strong_), (2) a weak T7 promoter (P_T7,weak_), (3) a strong σ^70^ promoter (P_σ^70^,strong_), or (4) a weak σ^70^ promoter (P_σ70,weak_). Relative strengths of the promoters are shown in Figure S1. The use of promoters compatible with two different RNA polymerases—the *E. coli* and T7 RNA polymerases (T7 RNAP), which were both present in the lysate used for the cell-free reactions—allowed more detailed analysis of resource limitations.

The “empty” or minimal plasmids do not express a reporter gene but were also designed to allow more detailed analysis of resource limitations. We refer to these plasmids as (1) “Empty”, with no promoter driving reporter gene expression; (2) “EmptyT7”, with a P_T7,strong_ promoter; and (3) “EmptySigma70”, with a P_σ70,strong_ promoter. EmptyT7 and EmptySigma70 each transcribe a 51 nt sequence from their respective promoters that is not subsequently translated; this sequence is immediately followed by a T7 or Sigma70 terminator, respectively. However, none of these vectors are truly empty: as required for plasmid growth and maintenance, all contain the same origin of replication (ori) and gene encoding antibiotic resistance, which lead to transcription and/or translation and thus may contribute to plasmid crosstalk.

**Figure 1:**
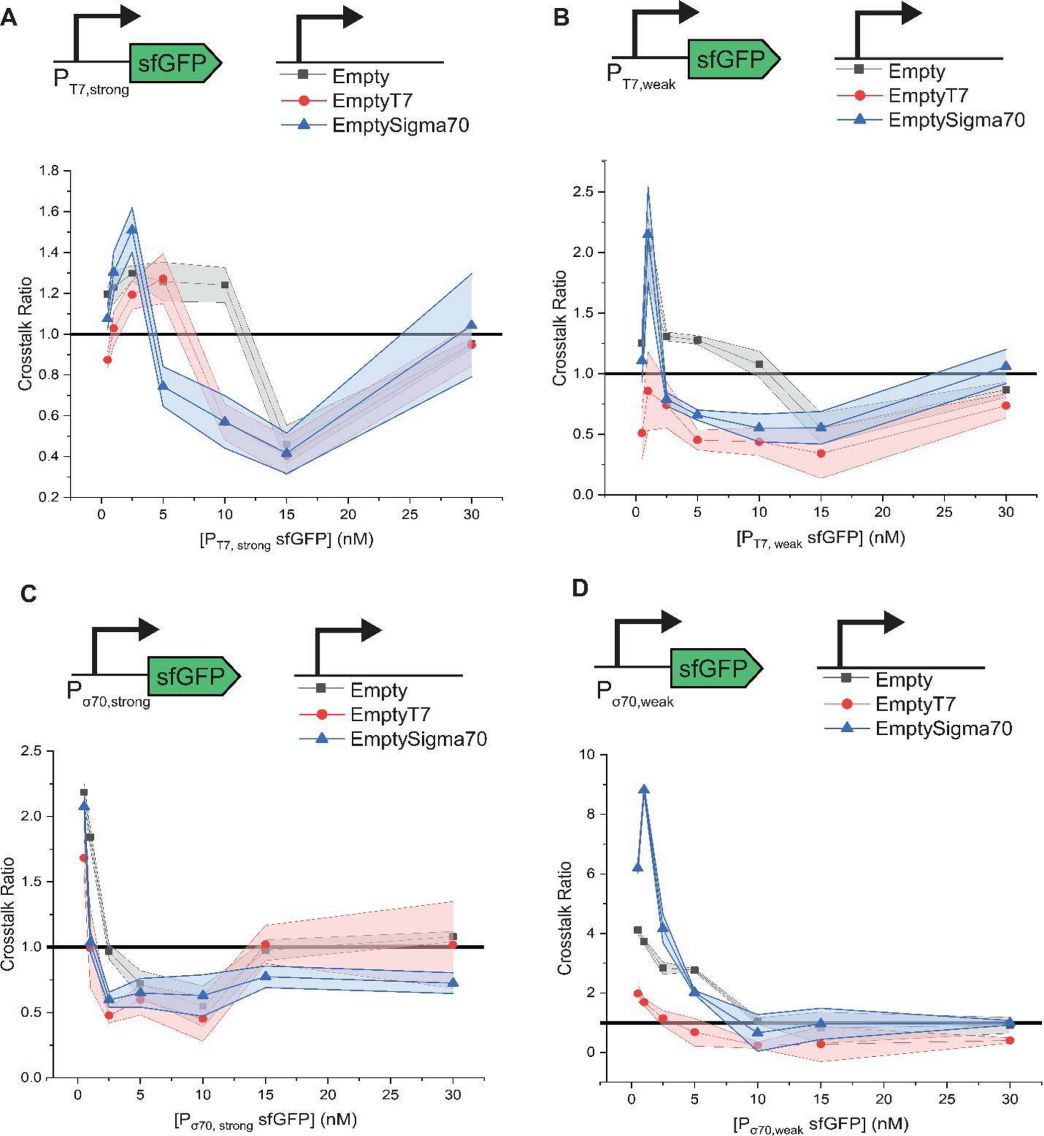
Assessment of plasmid crosstalk for protein reporters in a T7 RNAP-enriched lysate. Shown are the effects of addition of Empty, EmptyT7, or EmptySigma70 vectors on sfGFP levels transcribed from one of four promoters: (A) P_T7,strong_, (B) P_T7,weak_, (C) P_σ70,strong_, and (D) P_σ70,weak_. Addition of an empty vector changes reporter protein expression levels in a fashion dependent upon the reporter’s promoter, the empty vector’s promoter (or lack thereof), and the concentration of the reporter plasmid. In all subpanels, data were collected after a 3 h incubation at 37 °C, and background fluorescence was subtracted. The shaded region indicates the standard deviation of technical triplicates. Time course data for this experiment are included in Figure S3.

Changes to reporter levels were observed in all promoter and empty vector combinations under at least some conditions, demonstrating how prevalent plasmid crosstalk is (Figure 1; raw expression curves are shown in Figure S2). We observed both positive and negative crosstalk, with the quantitative levels of crosstalk appearing to be a function of promoter strength, reporter plasmid concentration, and empty vector contents.

At lower reporter plasmid concentrations, almost all promoter and empty vector combinations exhibit positive crosstalk. The maximum extent of these effects appears to be negatively (though not perfectly) correlated to the strength of the promoter in the reporter plasmid. This trend could be the result of RNase activity being distributed no longer across just the reporter transcripts but also across the noncoding or resistance marker transcripts, leading to extended mRNA half-life for the reporter gene—a phenomenon we refer to as “nuclease distraction”. Reporter constructs with weaker promoters like P_σ70,weak_ generate fewer mRNA transcripts to begin with, and thus benefit more from the presence of a relatively larger pool of transcripts to distract nucleases. In support of this theory, the combinations of reporters and empty vectors that exhibit positive crosstalk are often correlated with an increase in the apparent rate of sfGFP production at early time points (Figure S3), and not just a similar rate of production over a longer period of time; this increase in apparent rate is consistent with increased persistence of transcripts, enabling greater protein expression. However, in some cases, the maximum extent of positive effects is not at the lowest concentration of reporter DNA tested. This observation could indicate there is a minimum number of transcripts that must be produced from the reporter plasmid to maximize the positive effects of nuclease distraction (though the mechanism by which this would occur is not evident), or it could be evidence of some separate and yet to be determined phenomenon.

A prominent exception to this trend is the negative crosstalk when EmptyT7 is present at low reporter concentrations of P_T7,weak_ and at the lowest concentration of P_T7,strong_. One possible explanation for this exception could be the competition for T7 RNAP outweighing the positive crosstalk of nuclease distraction, which is consistent with the larger reporter plasmid range leading to negative effects and the greater magnitude of negative effects when using a weaker promoter due to the decreased affinity for polymerases compared to the empty vector. Accordingly, this trend is exacerbated for P_T7,weak_ when the same experiment is done in a cell lysate with basal (rather than induced) levels of T7 RNAP, as evidenced by the greater magnitude of negative effects at low reporter plasmid concentrations compared to the induced lysate (Figure S4). Time-course measurements in basal lysate also show a decrease in the apparent rate of sfGFP production for these cases rather than a shorter production period, further supporting this hypothesis (Figure S5).

At moderate concentrations of reporter plasmids with P_T7,weak_, P_T7,strong_, and P_σ70,strong_, negative effects are observed for multiple empty plasmids but not always all empty plasmids. This suggests that the negative effects are not due to polymerase competition but rather due to a more nonspecific phenomenon that is directly linked to transcription since these negative effects are often least prominent in the Empty vector. Interestingly, these negative effects are seemingly not correlated with a decrease in sfGFP production rate at early time points but instead appear to be an early cessation of sfGFP production (Figure S3). A possible explanation for this is toxic product buildup due to a maximum possible total expression capacity. A decrease in protein production at high P_T7,weak_, P_T7,strong_, and P_σ70,strong_ reporter plasmid concentrations without any empty vectors support this hypothesis (Figure S2). This trend has been described previously,^8^ though its potential role in the context of crosstalk has not previously been addressed, and the exact mechanism is not well understood.^19−21^ Toxic product buildup due to high levels of transcription may occur at lower plasmid reporter levels via the addition of a second plasmid. This theory is further supported by EmptyT7 and EmptySigma70 (with their additional 51-nt transcript), inducing negative effects at lower reporter concentrations compared to Empty. In further support of this theory, EmptySigma70 induces negative effects at lower concentrations than Empty T7 when the reporter plasmid is transcribed from P_T7,strong_ (Figure 1A). This trend cannot be fully explained by competition for resources, as EmptySigma70 does not compete with the P_T7,strong_ plasmid for polymerases.

The weakest promoter tested, P_σ70,weak_, exhibits a different behavior. It never reaches maximal protein expression (Figure S2) with Empty, EmptySigma70, or the baseline, as toxic product buildup may never occur due to the lower levels of transcription; this likely explains the lack of negative crosstalk (Figure 1). However, EmptyT7 expression still leads to negative effects (Figures S2 and 1) in a way that appears to be dependent on T7 polymerase levels as these negative effects are not seen in a lysate with basal T7 (Figure S4). Taken together, these observations support the hypothesis that stronger empty vector expression brings the system closer to toxic transcription levels that cause negative crosstalk.

At high reporter plasmid concentrations (30 nM), most combinations of reporter plasmid and empty vector yield reporter levels insignificantly different from those of the baseline, denoting no crosstalk. This could be attributable to the impacts of toxic expression levels eventually reaching a maximum, supported by the fact that the raw expression for P_T7,weak_, P_T7,strong_, and P_σ70,strong_ at the highest reporter levels is the same with or without empty vectors (Figure S2). For P_σ70,weak_ with nontoxic levels of expression, neutral plasmid crosstalk is likely due to higher mRNA levels buffering the reporter from positive crosstalk.

### Transcriptional Crosstalk

To decouple any potential translational crosstalk effects due to the resistance marker, we next used a fluorescent RNA reporter to characterize crosstalk. Specifically, we used the 3-Way Junction dimeric-Broccoli (3WJdB) RNA aptamer,^22^ which fluoresces when a dye is added to the reaction. We cloned it under control of P_T7,strong_, P_T7,weak_, and P_σ70,strong_ (P_σ70,weak_ was not used because its transcription was too weak for reliable measurement; data not shown). We recapitulated the experimental design from Figure 1, except with the reporter plasmid expressing the aptamer. Since RNA levels do not plateau like protein levels do in our cell-free system (due to the use of protease-deficient *E. coli* for lysate preparation), it was necessary to measure time-course, rather than single-end point, fluorescence for these experiments.^23^ Both positive and negative crosstalk were apparent at the transcriptional level across lysate batches (Figure S6), though the RNA-level trends did not directly match with those observed at the protein level, pointing toward the existence of multiple causes for crosstalk.

**Figure 2:**
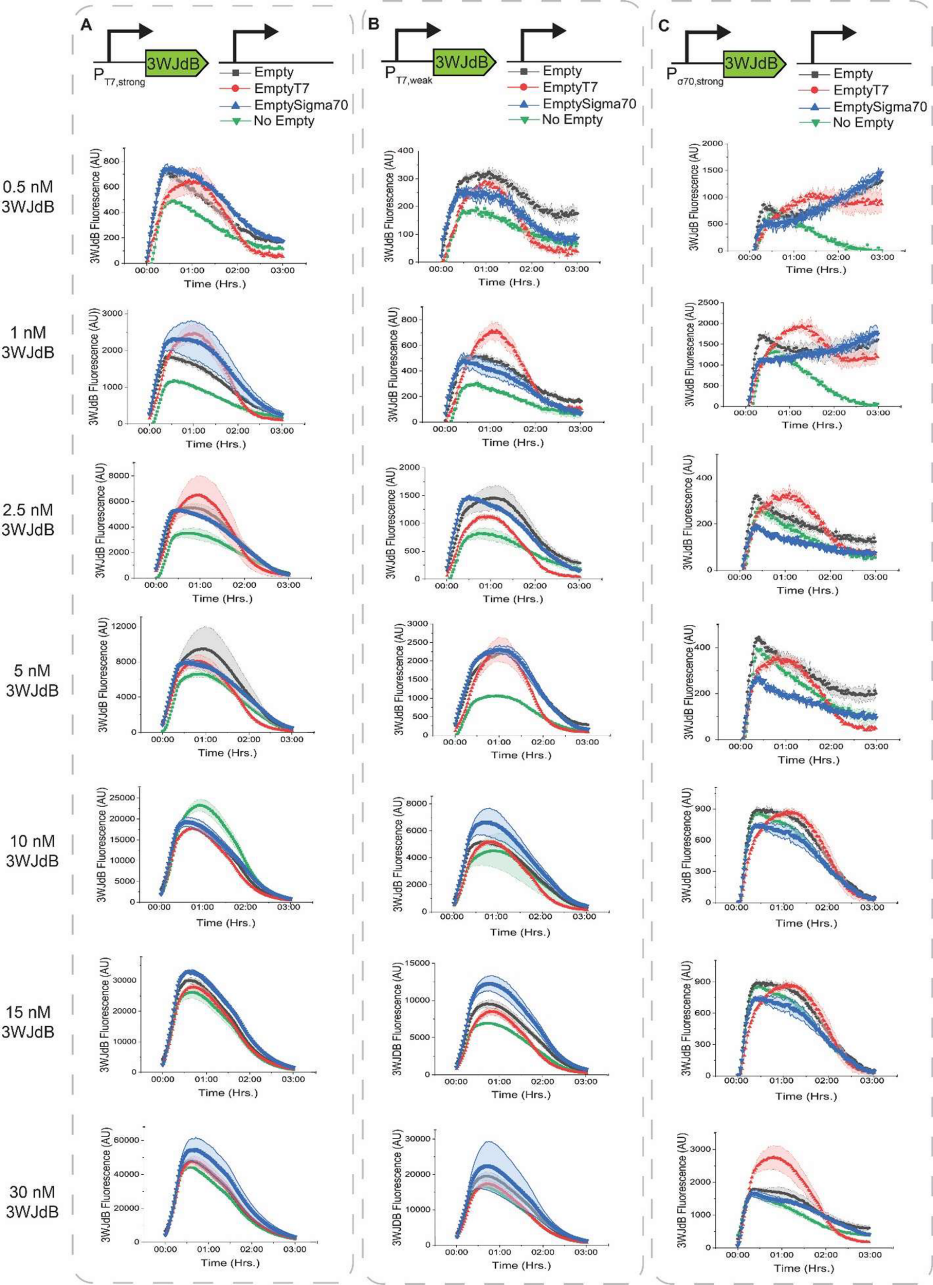
Assessment of plasmid crosstalk at the mRNA level in a T7-enriched lysate. Shown are the effects of the addition of different empty vectors on 3WJd-Broccoli RNA levels transcribed from one of three promoters: (A) P_T7,strong_, (B) P_T7,weak_, or (C) P_σ70,strong_. Curves above the green “No Empty” curve indicate positive crosstalk, while curves under the “No Empty” curve indicate negative crosstalk. Addition of an empty vector can cause substantial changes in reporter expression levels and the shapes of expression curves. Data were processed as described in Figure S7. In all subpanels, reactions were run for 3 h at 37 °C, and background fluorescence was subtracted. The shaded region indicates the standard deviation of technical triplicates.

When the RNA reporter is transcribed from a T7 promoter (Figure 2A and B), at low reporter plasmid concentrations, crosstalk is positive in almost all cases, as evidenced by the green curve with no empty vector being the lowest throughout the experiment. At high reporter concentrations, these effects are minimized, consistent with Figure 1. However, the concentration at which crosstalk transitions from positive to negative or neutral and the extent of these effects depend on the type of empty vector, the reporter promoter strength, and the lysate batch (Figure S6A and S6B). Unexpectedly, strong negative effects are minimal, suggesting that polymerase competition and toxic product build-up are less prevalent in this regime, possibly due to the smaller transcript size in the reporter vector and/or the lack of translation.

One exception to this trend occurs when any additional vector is added to 10 nM of P_T7,strong_ (Figure 2A) resulting in negative crosstalk. Though this phenomenon may be due to the negative effects of toxic product build-up outweighing the positive effects of nuclease distraction, we note that the toxic transcription hypothesis is likely not the only cause; otherwise, we would not expect to see greater total transcription at the higher reporter plasmid levels. This observation suggests the possibility of an additional currently unknown mechanism for negative transcriptional crosstalk.

Crosstalk with the P_σ70,strong_ promoter (Figure 2C and Figure S6C), on the other hand, exhibits different trends. For example, EmptySigma70 induces negative crosstalk at moderate reporter DNA concentrations, likely due to polymerase competition. Also, the addition of EmptyT7 can induce positive crosstalk at high reporter plasmid concentrations, which is counterintuitive because the effects of nuclease distraction should be less significant in this regime.

Crosstalk seemingly has a strong effect on the kinetics of mRNA expression, as evidenced by changes in the shapes of the mRNA expression curves. Most curves exhibit an initial rise, a local maximum, and then a decline in reporter levels, mediated by the ratio of the rate of transcription compared to that of RNA degradation as transcription gradually ceases. However, the time of the reporter maximum appears to be affected by plasmid crosstalk. For a *P*_σ70,strong_ reporter, addition of the EmptyT7 plasmid uniquely delays the time of the maximum output for many reporter plasmid concentrations. Moreover, some profiles exhibit a fast peak and slow decay, while others have more parabolic character. In one lysate and a set of conditions, the addition of all empty vectors led to a loss of the typical kinetic curve, indicating persistent transcription; this observation was reproduced across multiple days of experiment but not in the second lysate, and its cause is not immediately evident.

### Mutual Plasmid Crosstalk

In practice, plasmid crosstalk is often observed as a change in the levels of a single, measurable reporter protein (e.g., GFP) of interest, but it affects—to different degrees—all proteins expressed in the reaction. When the relative levels of each protein in the system matter, this becomes a critical consideration. Accordingly, we next assessed crosstalk between two reporter proteins, sfGFP and monomeric red fluorescent protein (mRFP). This experiment not only allows us to explore “mutual” crosstalk but also provides some insight into translational effects as the second plasmid is no longer an “empty” vector but instead expresses another reporter protein. In addition, it also lets us see more than just a binary presence or absence of a second plasmid but also a dose response of one plasmid (expressing mRFP) as the other plasmid (expressing sfGFP) has its concentration increased.

**Figure 3:**
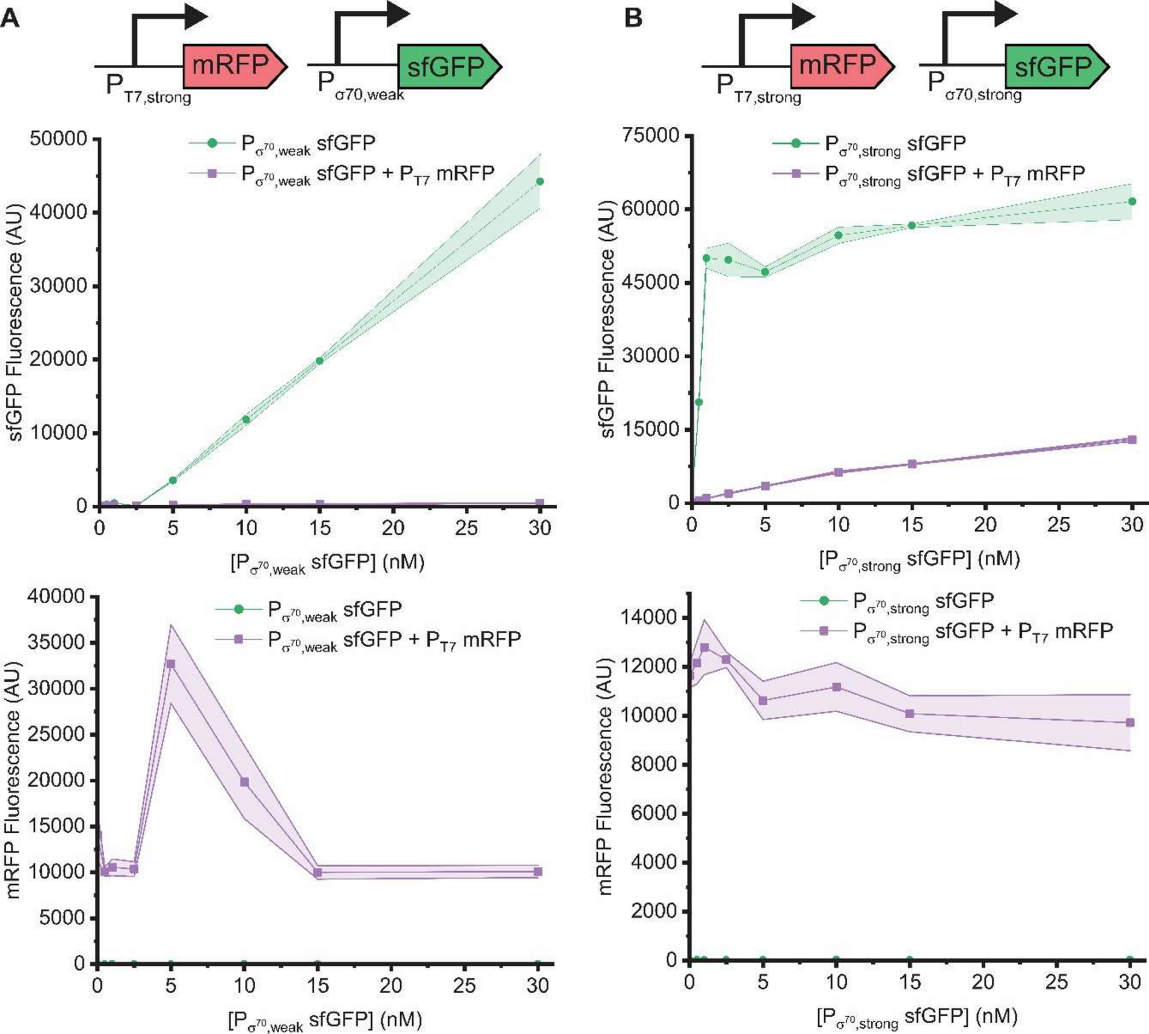
Mutual plasmid crosstalk in a T7 RNAP-enriched lysate. (A) Coexpression of GFP from P_σ70,weak_ and RFP from P_T7,strong_. Top: GFP levels decrease significantly upon expression of RFP from a strong promoter (with curve barely distinguishable from the *x* axis). Bottom: GFP coexpression has a positive effect on RFP levels within a range of GFP plasmid concentrations. (B) Coexpression of GFP from P_σ70,strong_ and RFP from P_T7,strong_. Top: Again, GFP levels decrease upon expression of RFP but to a lesser extent. Bottom: When GFP is also expressed from a strong promoter, RFP levels generally decrease with increasing concentrations of the GFP plasmid, and positive crosstalk is no longer observed. In all subpanels, the RFP-expressing plasmid was added at 10 nM. Data were collected after 4 h of incubation at 37 °C. The shaded region indicates the standard deviation of technical triplicates.

Coexpression of GFP from P_σ70,weak_ with RFP from P_T7,strong_ led to substantial negative crosstalk effects on GFP but some positive crosstalk effects on RFP. GFP levels (which did not reach saturation even at the highest plasmid concentration tested, likely due to the weak promoter driving its expression) were reduced by orders of magnitude by addition of a constant concentration of the RFP plasmid, although they still increased with increasing concentrations of the GFP plasmid (Figure 3A, top). Comparison of these results to Figure 1D, where positive crosstalk is observed at low GFP plasmid concentrations, suggests that this significant negative crosstalk must primarily occur at the level of translation: the very strong T7 promoter quickly generates many RFP transcripts that likely monopolize the ribosomes in the reaction, resulting in lower GFP protein expression in this experiment but not in Figure 1D where the T7 transcripts are not translated. RFP levels increase within a certain range of GFP plasmid concentrations (Figure 3A, bottom), likely due to nuclease distraction by GFP transcripts. As previously seen, protein expression is more robust to plasmid crosstalk when transcription of that protein is driven by a strong promoter, and the second protein is driven by a weak promoter, a strategy that can be used to minimize negative effects on the expression of a target protein that is needed at higher titers.

When GFP was instead expressed from P_σ70,strong_, the crosstalk effects on both GFP and RFP were markedly different. While GFP levels still decreased substantially, the reduction was nowhere near as great as when GFP was expressed from P_σ70,weak_ (Figure 3B, top). Also in contrast to the P_σ70,weak_ case, RFP levels no longer exhibited positive crosstalk (Figure 3B, bottom); instead, they decreased slightly with increasing concentrations of the GFP plasmid, potentially the result of ribosomal competition. Taken together, these experiments show that each plasmid in a cell-free reaction could potentially have an impact on expression from the others and that the degree and type of crosstalk (positive or negative) depend on the strength of the promoter in each plasmid even if they use orthogonal transcriptional machinery.

### Plasmid Crosstalk in Common Cell-free Systems

Taken together, Figures 1, 2, and 3 demonstrate that crosstalk can cause considerable, yet unpredictable, changes to mRNA and protein output, which in turn can potentially lead to substantial confounding effects on the interpretation of experiments and data that otherwise seem straightforward. If one attributes changes when a plasmid is added to a system solely to the function of the product of the plasmid, incorrect conclusions can easily be reached. To demonstrate this possibility, we present two simple systems where plasmid crosstalk can lead to results that could otherwise be misinterpreted if crosstalk is not properly considered.

**Figure 4:**
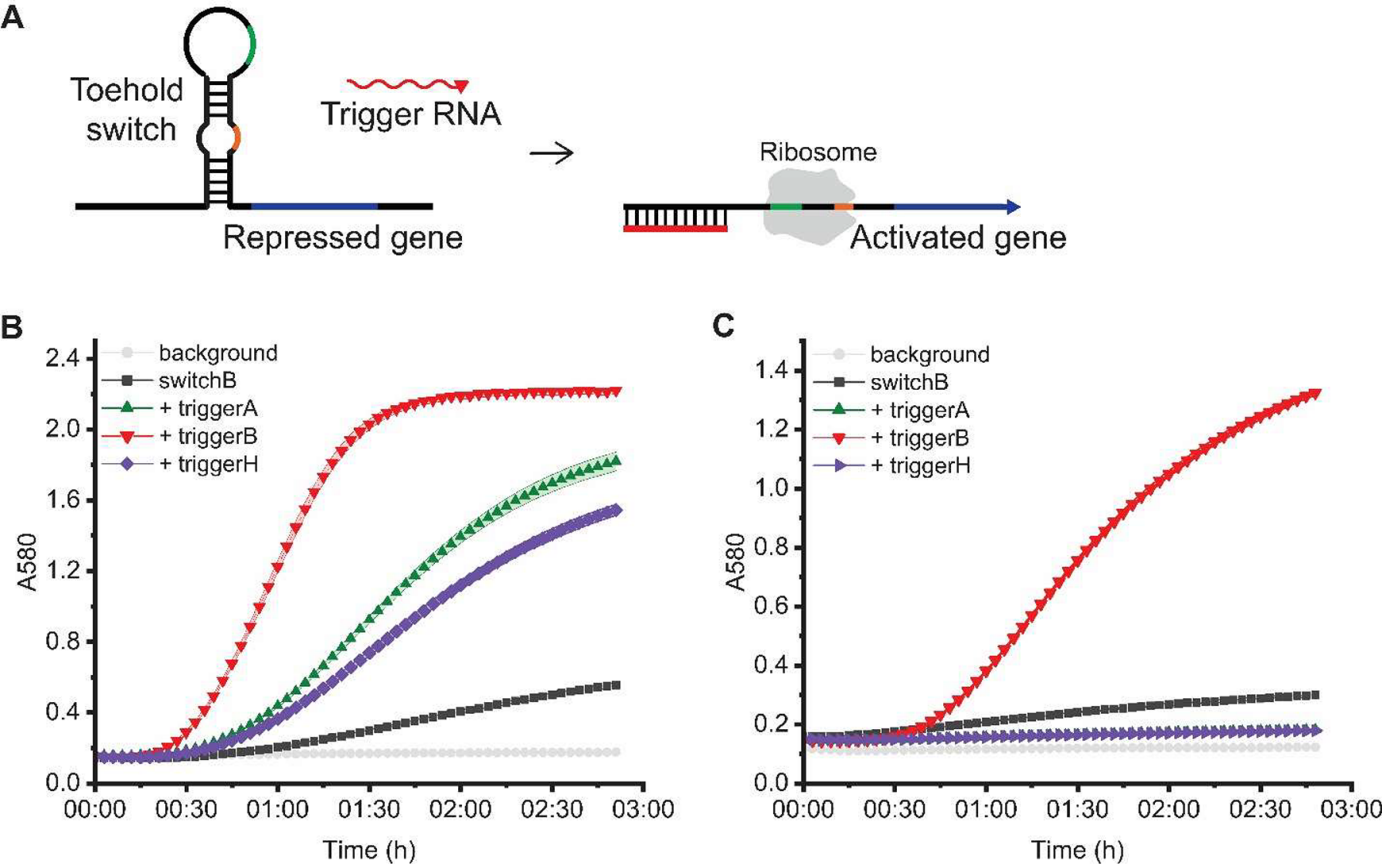
Effects of plasmid crosstalk in a toehold switch system. (A) Translational regulation by a toehold switch. In the absence of the trigger RNA, the toehold switch secondary structure occludes the RBS, preventing translation. Binding of the trigger RNA to a recognition sequence in the toehold switch exposes the RBS to ribosomes, allowing translation. (B) Toehold switch B response to three plasmid-borne triggers: A, B, and H. Even though only trigger B should activate switch B, expression of all three triggers induces β-galactosidase production beyond background levels, resulting in β-galactosidase-mediated catalysis and an increase in absorbance values. (C) Response to 2 μM RNA triggers. Only trigger B leads to a significant increase in absorbance values. In all subpanels, reactions were run in a lysate with basal levels of T7 RNAP and incubated at 37 °C. The shaded region indicates the standard deviation of technical triplicates.

First, we considered a simple system driven by a toehold switch, a riboregulator that has been widely used in cell-free biosensors.^24−26^Figure 4A depicts the general regulatory mechanism of toehold switches: RNA secondary structure in an inactivated toehold switch blocks the translation of a gene by occluding the RBS, but in the presence of a trigger RNA with complementarity to a specific section of the switch, it undergoes a conformational change that exposes the RBS and allows translation of the gene product. For this example, we used a previously characterized toehold switch (switch B) to control expression of β-galactosidase (LacZ), which cleaves a yellow molecule to form a purple product that results in an increase in absorbance at 580 nm. This switch was paired with its corresponding trigger (trigger B) as well as two orthogonal triggers (A and H)^27^ expressed from P_T7,strong_. Only trigger B should activate switch B.

Expressing the triggers from plasmids leads to what may initially seem to be unexpected results. As expected, the expression of trigger B from a plasmid leads to activation of the toehold switch (Figure 4B). However, expression of triggers A and H from plasmids also leads to activation, though at a lower level, potentially suggesting off-target effects from what should be orthogonal triggers.

Directly adding RNA triggers along with RNase inhibitors yields results that are what otherwise would have been expected without plasmid crosstalk. Only RNA trigger B activates switch B (Figure 4C), suggesting that the activation observed when triggers A and H were expressed from plasmids was not due to them physically interacting with switch B. The most likely explanation for the apparent activation seems to be positive crosstalk due to nuclease distraction.

Avoiding misinterpretation of experimental results due to plasmid crosstalk in this toehold switch system thus requires careful experimental design. As shown in Figure 4C, RNA triggers can be used instead of plasmid-borne triggers. However, using RNA triggers requires an *in vitro* transcription step to generate these transcripts that may not scale well to screens of many triggers and switches. It also requires the addition of expensive RNase inhibitors to the reaction. Using linear DNA instead of plasmids or RNA may help reduce crosstalk via elimination of transcription and translation from replication machinery and antibiotic resistance genes, but linear DNA is easily degraded in the lysate without protection against exonucleases^28^ and crosstalk effects from the trigger itself may still be present.

**Figure 5:**
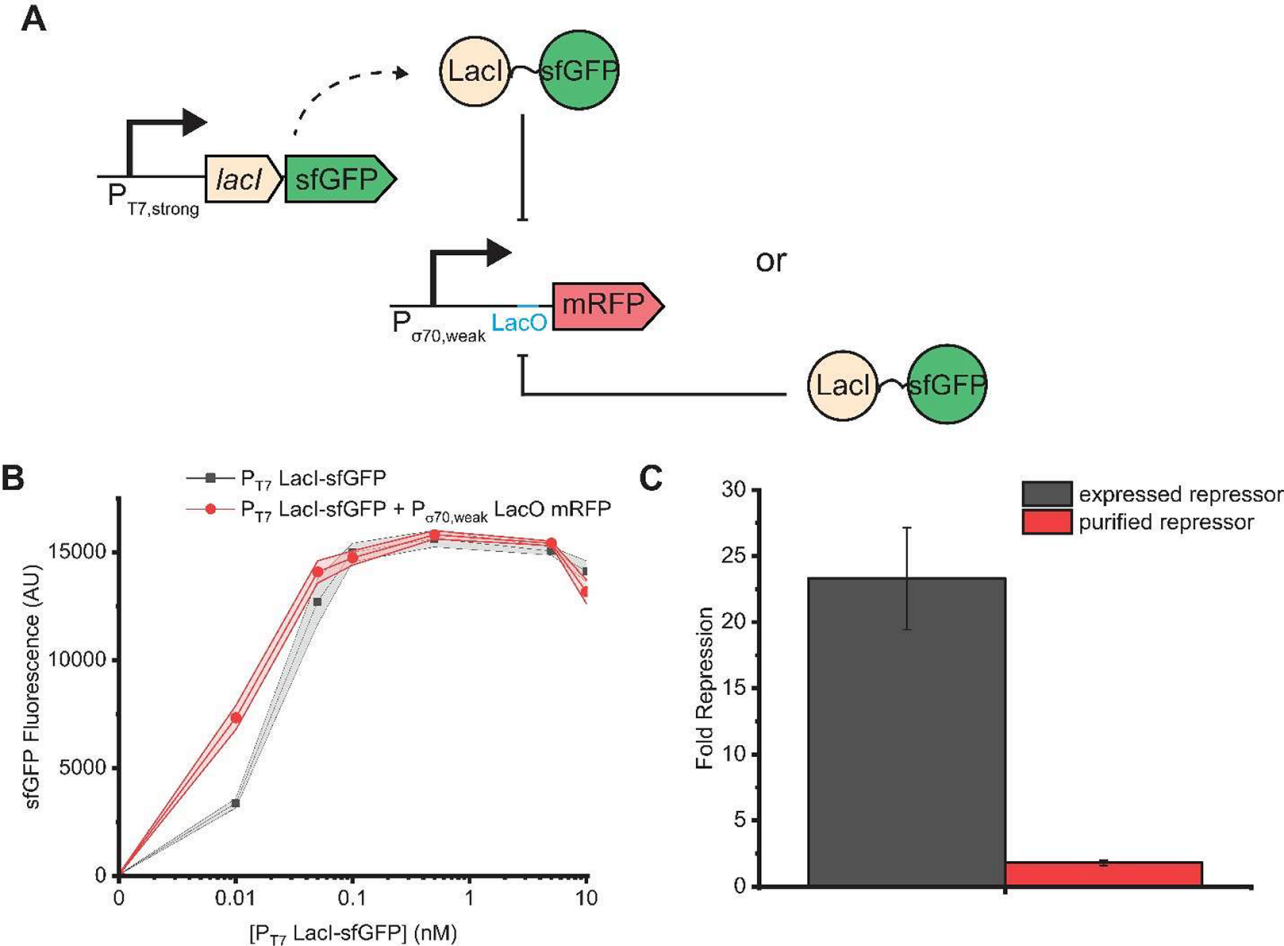
Negative crosstalk in a LacI repressor-based system. (A) Schematic of the LacI-based system. mRFP was expressed from a promoter containing the LacO operator, and a LacI-sfGFP fusion was added via either plasmid-based expression or direct addition of purified protein. (B) sfGFP fluorescence levels generated by plasmid-expressed LacI-sfGFP. Low plasmid dosage is sufficient to achieve maximum expression of LacI-sfGFP fusion. The addition of 10 nM of a plasmid expressing mRFP induces slight positive crosstalk at low concentrations of the LacI-sfGFP plasmid, but the expression profile is very similar overall. (C) Maximum fold repression of mRFP. The maximum reduction in RFP is 10 times greater with a plasmid-borne repressor than with a purified repressor, indicating that expression of the repressor from a plasmid introduces negative crosstalk that would otherwise be interpreted as repression directly mediated by LacI binding to LacO. In all subpanels, data were collected in a T7 RNAP-enriched lysate after 4 h of incubation at 37 °C. The shaded region indicates the standard deviation of technical triplicates.

To analyze further potential confounding effects of plasmid crosstalk, we next considered a simple LacI repressor-based system. A schematic depicting the design of this system is shown in Figure 5A. mRFP is expressed from a P_σ70,weak_ promoter regulated by the LacI repressor via its binding to the LacO operator sites; this construct is added to the reaction mixture as a plasmid. To assess the impact of negative crosstalk, LacI was added to the system either via expression from a plasmid using P_T7,strong_ or as a purified protein. In both cases, LacI was translationally fused with sfGFP so that LacI levels could be compared between the two. This type of regulatory circuit is used frequently in transcription factor-based cell-free biosensors.^29−31^

GFP levels are fairly robust to RFP coexpression (Figure 5B). There is slight positive crosstalk at low LacI-sfGFP plasmid concentrations, likely due to some nuclease distraction by RFP. The fluorescence levels at each concentration of plasmid were used as a basis for calculating the corresponding concentrations of purified LacI-sfGFP required to maintain similar levels of the repressor for comparison.

Analysis of the RFP levels for the plasmid-borne and equivalent purified protein concentration experiments revealed some surprising results. As expected, RFP levels decrease with an increase in LacI-sfGFP plasmid levels. Of note, though, multiple LacI-sfGFP plasmid levels that otherwise produced identical GFP fluorescence could lead to drastically different RFP levels (Figure S8). At the highest concentrations of the LacI-sfGFP plasmid, the repression of RFP expression was extremely strong. When purified LacI-sfGFP (SDS-PAGE gel shown in Figure S9) was used instead, the LacI-mediated repression was somewhat stronger at low repressor concentrations but much weaker compared to when high levels of repressor plasmid were used (Figure S8). Maximum repression of RFP expression was only approximately 2-fold by the purified repressor (potentially impacted by fusion with sfGFP) but approximately 20-fold by the plasmid-borne repressor (Figure 5C).

This difference in the extent of apparent repression by LacI suggests that a substantial portion of the repression observed when working with plasmid-borne repressors may, in fact, be due to negative plasmid crosstalk. These negative effects could arise from translational resource competition; *lacI-sfGFP* transcripts, generated from a strong promoter, likely monopolize translational machinery, resulting in even lower RFP levels. This negative crosstalk may go unnoticed in the development of regulatory circuits, as it could just be attributed to the expected function of a repressor. However, the repressor would actually not be as strong as it appears, with limited dynamic range for derepression.

### Mechanistic Basis of Plasmid Crosstalk

**Figure 6:**
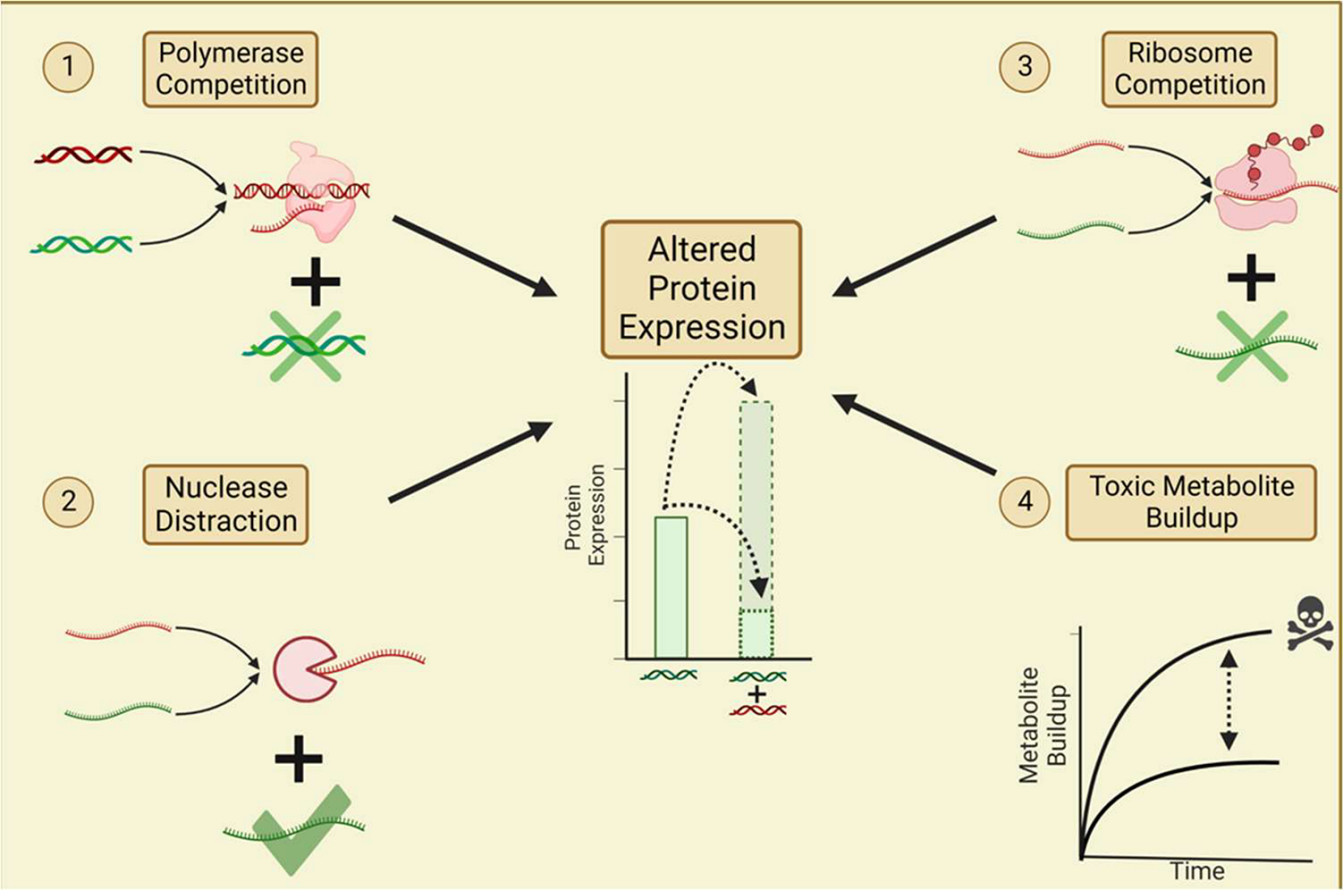
Proposed mechanisms for plasmid crosstalk. Addition of a second genetic cassette (shown in red) to a cell-free reaction can give rise to crosstalk for the reporter cassette (shown in green). Specifically, competition for RNA polymerases (1) and ribosomes (3) via the addition of a second cassette can lead to negative crosstalk. However, transcripts produced from the second plasmid may also serve as decoys for ribonuclease-mediated degradation, allowing more transcripts from the first plasmid to subsist in the lysate and generate higher protein levels (2). Transcription and translation both seem to produce toxic byproducts that accumulate and exacerbate negative crosstalk (4). Ultimately, crosstalk can lead to changes in the protein expression of the reporter cassette.^32^

Based on our data, we propose four potential mechanisms of plasmid crosstalk in CFE systems: RNA polymerase competition, ribonuclease distraction, ribosome competition, and toxic metabolite buildup (Figure 6). In each case, the degree and type of crosstalk—positive or negative—are affected by promoter strength and plasmid concentrations. This list is likely not exhaustive, as these mechanisms alone cannot explain all of the trends we have observed.

To further elucidate the effect of nuclease distraction, we assessed crosstalk in two systems: a crude lysate, which contains endogenous RNases, and PUREfrex, a purified enzyme-based CFE system that does not have any RNases. The addition of EmptySigma70 to a reaction expressing sfGFP from P_T7,weak_ does not alter protein levels in PUREfrex but slightly increases them in the crude lysate (Figure S10). Since crosstalk is observed only in the crude lysate, it is likely that mRNA decoys play a role in generating positive crosstalk. This is one example of an experiment that can provide more insights into factors that potentially contribute to crosstalk; additional experiments are needed to flesh out the proposed mechanisms and identify other mechanisms.

## Conclusion

Crosstalk can have positive or negative impacts on both RNA and protein levels. These effects are a function of the promoter strength, plasmid concentrations, and transcriptional machinery used for the corresponding promoters. We believe that ribonuclease distraction, toxic metabolite buildup, and competition for transcriptional and translational resources are key mechanisms for generating crosstalk, but additional mechanisms likely exist that have yet to be elucidated. While these effects may at first seem to be oddities or artifacts with little impact, we demonstrated with multiple examples that crosstalk can confound the interpretation of results from cell-free expression networks. This in turn complicates the design and implementation of complex cell-free circuits and reaction networks, lengthening the design–build–test–learn cycle at a time when the field is heavily invested in shortening that cycle.

Perhaps the biggest challenge to fully elucidating plasmid crosstalk is our incomplete understanding of CFE systems. For example, although they are far from a “black box”, there remains a need to thoroughly investigate the underlying phenomena that make cell-free gene expression still often unpredictable and unreliable, such as its inherent batch-to-batch variability. Moreover, existing computational models of cell-free systems are still unable to capture some of the key phenomena we have characterized here. A recently developed CFE *in silico* model^33^ that accounts for resource competition and even explicitly models nucleases was unable to recapitulate the crosstalk data seen in Figure 2, even when given a 100-fold parameter search space around (10-fold above and 10-fold below) the default and literature parameters (Figure S11). This supports the importance of developing computational models that are predictive of known and observable CFE phenomena.

Another example of incomplete understanding worth noting is that the cell lysate has active endogenous metabolism that causes substantial changes in metabolite levels in cell-free reactions which lead to reduced protein-production capacity independent of expression from a plasmid.^34,35^ This endogenous metabolism may interact with or even contribute to toxic metabolite buildup associated with gene expression, which itself can be variable across constructs. Understanding the relationship between gene expression and toxic metabolite buildup is critical for predicting crosstalk.

Nonetheless, qualitative trends caused by the proposed crosstalk mechanisms are consistent across lysate batches, even if the quantitative details may vary. Interbatch and interoperator variation in lysate performance characteristics is a widely known issue for crude lysate-based CFE systems.^36^ As a result, the characteristics of a lysate can sometimes affect the observed function of genetic programs implemented in cell-free reactions by using those lysates. Here, though, we observed consistent trends across lysates prepared by different researchers and, in most cases, with different levels of T7 RNAP. However, the exact plasmid concentrations at which specific types of crosstalk occur do vary from batch to batch.

Furthermore, we note that the phenomena that cause plasmid crosstalk affect any cell-free reaction and are not restricted to multiplasmid systems or even plasmid-based networks. We use the term “plasmid crosstalk” because in CFE systems there is often no good reason to assemble a single plasmid expressing multiple genes, but the conclusions would likely hold if all components of the genetic circuit were on the same plasmid or even expressed from a linear DNA template.

**Figure 7:**
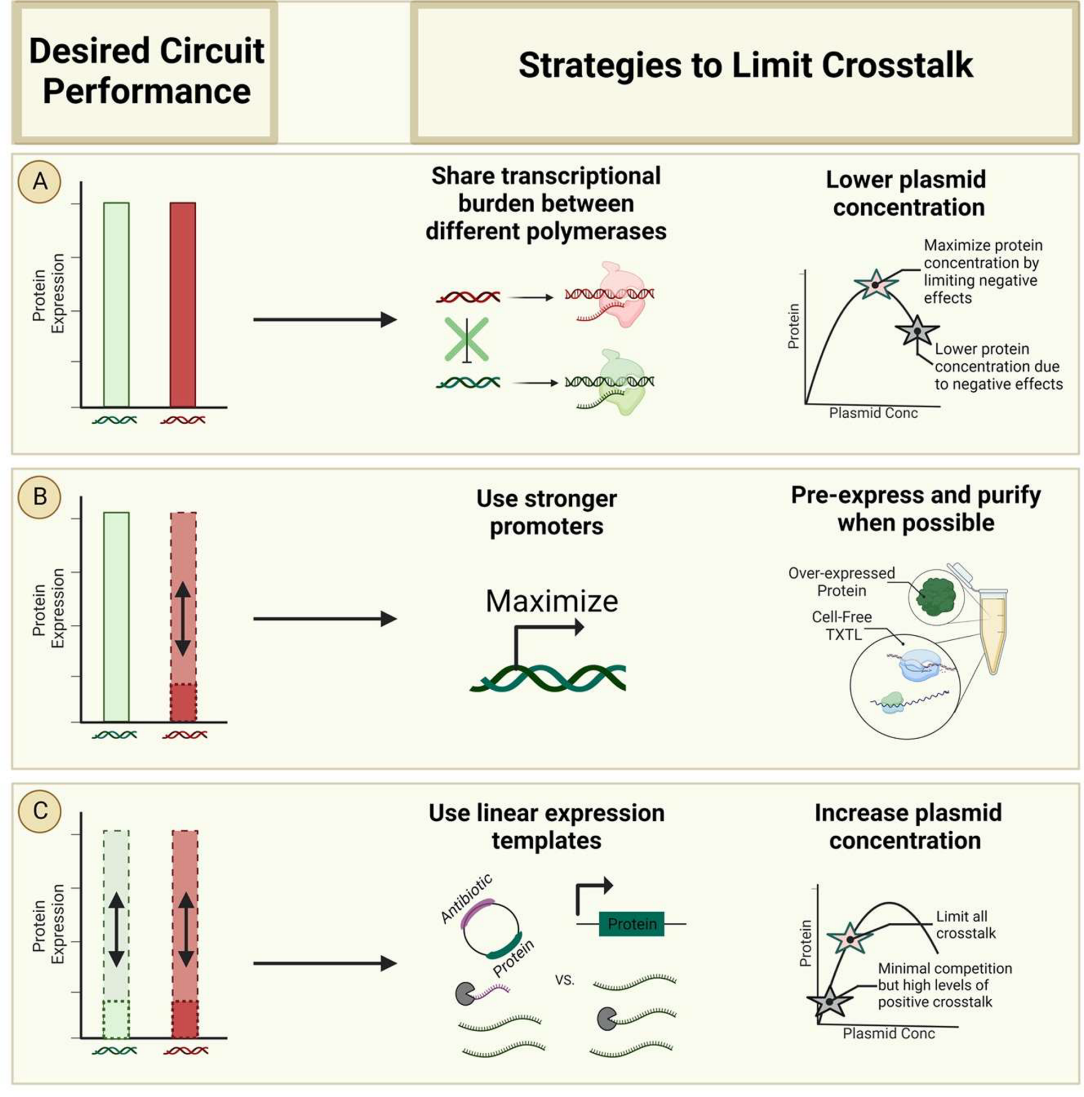
Strategies to limit adverse effects of crosstalk. (A) Strategies to minimize crosstalk when high final expression levels are desired for two different proteins. Negative crosstalk due to transcriptional competition can be decreased by using promoters corresponding to different polymerases (e.g., T7 vs native *E. coli* RNAP with σ^70^). Additionally, maximum final expression levels can be achieved by using midlevel plasmid concentrations to reduce negative effects from toxic product accumulation. (B) Strategies to minimize crosstalk when a very precise level of one specific protein is needed even as other components may vary in concentration. We recommend using strong expression (promoter and RBS) on the plasmid coding for the protein that needs to be constant to decrease the extent of crosstalk generated by other genetic cassettes in the reaction. Alternatively, proteins can be added directly in purified form, from pre-expression in a separate cell-free reaction or via lysate enrichment. (C) Strategies to minimize crosstalk when multiple proteins are varying in concentration. Positive crosstalk due to nuclease distraction can be limited via use of expression templates that do not include antibiotic resistance markers or origins of replication (e.g., linear DNA). Resource competition can be limited by working at low plasmid concentrations (though potentially resulting in high levels of positive crosstalk). Alternatively, we recommend using midlevel plasmid concentrations, limiting both negative crosstalk due to toxic product buildup and positive crosstalk due to nuclease distraction.^32^

The design and implementation of genetic circuits that require precise tuning for their function can be confounded by the effects of crosstalk, meaning that laying out some design principles may be useful for enabling more robust development. Looking at the entirety of our findings and their context within existing literature reports, we suggest a few strategies that can be applied to limit the adverse effects of plasmid crosstalk (Figure 7A–C).

Some genetic circuit designs might require high final expression levels of two different proteins, which can be challenging to implement, given the impacts of crosstalk. One way to address this challenge is via promoter selection. Transcriptional competition can be a significant contributor to negative crosstalk, so using promoters corresponding to different RNA polymerases (e.g., native *E. coli* versus T7 polymerase) to transcribe different genes will help distribute the transcriptional burden and decrease crosstalk (Figure 7A). While this strategy will not eliminate crosstalk at the translational level, it will at least help make the crosstalk simpler to manage and predict. Another approach to addressing this problem would be the moderation of the plasmid concentrations used for those two proteins. While the intuitive choice may be to use the highest possible concentrations of plasmid possible to maximize expression for each protein, this may lead to excessive negative crosstalk. Even if the transcriptional burden is distributed across two polymerases, it may lead to such high initial expression rates that toxic metabolite accumulation can cause early cessation of expression (Figure 7A). To this end, choosing a more moderate level of plasmid concentration provides an opportunity to limit negative crosstalk and yield higher expression.

Other circuit designs may not require high levels of multiple proteins but may require a very precise level of one specific protein even as other components may vary in concentration. One strategy to achieve this goal would be to have rather strong expression (promoter and RBS) of the plasmid coding for the protein that needs to be constant and relatively low concentrations of other plasmids (Figure 7B). Our data suggest that in some regimes, there is a negative correlation between promoter strength and extent of crosstalk. One contributor to this correlation is likely that the effects of transcription and translation from stronger promoters mask the impacts of the other components. Therefore, we suggest (when possible) the use of relatively strong promoters to achieve high titers of a target protein and to reduce potential crosstalk. With sufficiently high concentrations of the first plasmid, the chances for positive crosstalk will be low.

However, if that protein’s constant concentration must be fairly low, this can be quite challenging, as positive or negative crosstalk will likely affect the levels of that protein. In this case, adding these proteins to the cell-free system instead of expressing them in the cell-free reaction can be an effective strategy (Figure 7B). The most direct way of achieving this would be to add purified proteins, although this may not always be practical or economical. Alternatives that may entail lower cost or effort include enrichment^31^ of the lysate via creation of an overexpressing background strain for lysate preparation or pre-expression^37^ of a protein in a separate cell-free reaction used to supplement the final cell-free reaction.

Last, more complex circuit designs may involve regulatory interactions that result in varying levels of multiple proteins over the course of the cell-free reaction or under different environmental conditions. In these regimes, crosstalk can be difficult to avoid. To begin to limit the impacts of crosstalk, we recommend the use of expression templates that do not contain an antibiotic marker or an origin of replication, such as linear DNA (Figure 7C). As noted above, even “empty” vectors that contain only these components can show significant crosstalk, so eliminating their presence will simplify the consideration of crosstalk impacts. However, currently the use of linear DNA in lysate-based cell-free systems is not simple and is in some cases not feasible; therefore, this may not always be an option.

Additionally, judicious selection of total plasmid concentration can help limit the potential impact of crosstalk in this regime (Figure 7C). Previous work has described the use of low concentrations of plasmids as a generalized strategy to minimize crosstalk, but those recommendations considered only negative crosstalk.^8^ The use of very low plasmid concentrations may itself not always be feasible for a given application as it can decrease protein titer to unacceptable levels. However, even when feasible, extremely low plasmid concentrations are more likely to lead to positive crosstalk, which can have equally confounding effects on the implementation of a precisely tuned circuit. Instead, we suggest using midrange plasmid concentrations to avoid negative crosstalk at high plasmid concentrations (perhaps due to toxic product buildup) as well as positive crosstalk at low concentrations due to nuclease distraction.

On the other hand, with careful consideration of genetic circuit design and reaction conditions, crosstalk can instead be deliberately exploited as a functional feature. For example, a transcription factor-based biosensor can be designed to maximize negative crosstalk and thus reduce background expression, which is an important figure of merit for some applications, including point-of-care deployment.^30^ Alternatively, the production of a target protein could potentially be increased by adding a second plasmid that will generate mRNA decoys for degradation.

Future investigations of plasmid crosstalk are expected to be critical in advancing our understanding of this poorly characterized phenomenon. One important focus should be on identifying the metrics and measurements that would sufficiently characterize cell-free lysates to allow for incorporation of nuclease degradation, resource competition, and toxic metabolite buildup into mathematical models of CFE. Cell lysates are not defined mixtures, and the apparent strength of different lysate batches for different expression machinery can vary significantly, making a model capable of quantitatively predicting plasmid crosstalk currently impossible. Moreover, the field would greatly benefit from studies that reveal additional factors that affect transcription and translation in cell-free reactions, helping to reconcile some of the crosstalk data that are not adequately explained by the mechanisms described here. Gaining a better understanding of the complex causes and impacts of plasmid crosstalk on protein expression is an important step toward enabling efficient and reliable implementation of genetic circuits in CFE systems.

## Methods

### Materials

T4 DNA ligase, T5 exonuclease, Taq ligase, Phusion polymerase, Q5 polymerase, and restriction endonucleases were purchased from New England Biolabs (Ipswich, MA, USA). E.Z.N.A. Plasmid Mini and Midi Kits were purchased from Omega Biotek (Norcross, GA, USA), and QIAquick PCR Purification Kits were purchased from QIAGEN (Valencia, CA, USA).

### Strains and Plasmids

*Escherichia coli* K12 DH10B (New England Biolabs, Ipswich, MA) was used for plasmid assembly. The plasmid pJL1, with a ColE1 origin and kanamycin resistance cassette, was used as the backbone vector for all plasmids used in cell-free experiments.

### Cloning and Construct Assembly

All constructs were assembled with inverse PCR or Gibson assembly.^38^ LB medium composed of 10 g/L NaCl, 5 g/L yeast extract, and 10 g/L tryptone was used for all cell growth during the cloning steps. Kanamycin (33 μg/mL) was used for appropriate antibiotic selection. The *sfGFP* gene was amplified from the plasmid pJL1, and *lacI* was amplified from previously constructed plasmids.^29^ The promoters P_σ70,strong_ (P_J23119_) and P_σ70,weak_ (P_J23105_) were obtained from the Anderson promoter collection in the Standard Registry of Biological Parts. All plasmid sequences are described in the Supporting Information.

### Preparation of Cellular Lysate

Unless otherwise specified, cellular lysate for all experiments was prepared as previously described.^39^ BL21 Star (DE3) *ΔlacZ* cells were grown in 2× YTP medium at 37 °C and 180 rpm to an OD of 1.7, which corresponded with the midexponential growth phase. Expression of T7 RNA polymerase was induced with 0.4 mM isopropyl β-d-1-thiogalactopyranoside (IPTG) around OD 0.6. Cells were then centrifuged at 2700 rcf and washed three times with S30A buffer, which contains 50 mM tris, 14 mM magnesium glutamate, 60 mM potassium glutamate, and 2 mM dithiothreitol, and is pH-corrected to 7.7 with acetic acid. After the final centrifugation, the wet cell mass was determined, and cells were resuspended in 1 mL of S30A buffer per 1 g of wet cell mass. The cellular resuspension was divided into 1 mL aliquots. Cells were lysed using a Q125 Sonicator (Qsonica, Newton, CT) at a frequency of 20 kHz and 50% of amplitude. Cells were sonicated on ice with three cycles of 10 s on and 10 s off, delivering approximately 300 J, at which point the cells appeared visibly lysed. An additional 4 mM of dithiothreitol was added to each tube, and the sonicated mixture was then centrifuged at 12 000 rcf and 4 °C for 10 min. The supernatant was removed, divided into 1 mL aliquots, and incubated at 37 °C and 220 rpm for 80 min. After this runoff reaction, the cellular lysate was centrifuged at 12 000 rcf and 4 °C for 10 min. The supernatant was removed and loaded into a 10 kDa MWCO dialysis cassette (Thermo Fisher). Lysate was dialyzed in 1 L of S30B buffer (14 mM magnesium glutamate, 60 mM potassium glutamate, 1 mM dithiothreitol, pH-corrected to 8.2 with Tris) at 4 °C for 3 h. Dialyzed lysate was removed and centrifuged at 12 000 rcf and 4 °C for 10 min. The supernatant was removed, aliquoted, and stored at −80 °C for future use.

Cellular lysate for the toehold switch and additional validation experiments was prepared as described above but was not induced with IPTG during cell growth.

### Cell-Free Reactions

Cell-free reactions for all experiments were run as previously described.^23^ Each cell-free reaction contained 0.85 mM each of GTP, UTP, and CTP, in addition to 1.2 mM ATP, 34 μg/mL folinic acid, 170 μg/mL *E. coli* tRNA mixture, 130 mM potassium glutamate, 10 mM ammonium glutamate, 12 mM magnesium glutamate, 2 mM each of the 20 standard amino acids, 0.33 mM nicotine adenine dinucleotide (NAD), 0.27 mM coenzyme-A (CoA), 1.5 mM spermidine, 1 mM putrescine, 4 mM sodium oxalate, 33 mM phosphoenolpyruvate (PEP), 27% cell extract, and plasmid concentrations specified for each experiment. Reaction with RNA triggers also contained RNase inhibitor (New England Biolabs) added to a concentration of 0.6 U/μL.

Reactions were run in 10 μL volumes in 384-well small volume plates (Greiner Bio-One), and a clear adhesive film was used to cover the plate and prevent evaporation. Plates were incubated for 3 or 4 h at 37 °C, and fluorescence was measured with a plate reader (Synergy4, BioTek). Time-course experiments were run directly in the plate reader. Excitation and emission wavelengths for sfGFP were 485 and 510 nm, respectively. Excitation and emission wavelengths for mRFP were 584 and 607 nm, respectively. Excitation and emission for 3WJdB were 472 and 507 nm, respectively. 3JDB RNA expression data were analyzed as described in Figure S7 to account for temperature effects on fluorescence levels, and negative values were discarded.

### PUREfrex Reactions

For experiments run with PUREfrex reactions (New England BioLabs), solutions I, II, and III were mixed as directed by the manufacturer. Plasmids were added at the concentrations indicated in Figure S10, and *E. coli* RNA polymerase holoenzyme (New England Biolabs) was added at 100 U/mL. Reactions were run in 5 μL volumes in 384-well small volume plates and incubated at 37 °C for 3 h. Fluorescence was quantified as previously described.

### Protein Purification

Plasmid coding for expression of his-tagged LacI-sfGFP (sequences provided in the Supporting Information) was transformed into BL21 (DE3) cells and plated on LB plates supplemented with 33 μg/mL kanamycin to grow overnight. One colony was selected the next day and resuspended in a 50 mL LB culture supplemented with 33 μg/mL kanamycin for overnight growth. The overnight culture was then diluted 100-fold in 500 mL of fresh 2× YTP media containing kanamycin the next morning and grown until its OD600 reached between 0.4 and 0.6, at which point 0.4 mM IPTG was added to induce T7 RNA polymerase expression and thus plasmid-driven protein production. The induced culture was transferred to a shaking water bath for incubation at 30 °C and 180 rpm for 16 h.

Cells were then centrifuged at 2700 rcf twice and washed with 1 × PBS. One gram of cells was resuspended in 2 mL of lysis buffer (50 mM Na_2_HPO_4_, 500 mM NaCl, 10 mM imidazole, pH 8). The resuspension was divided into 1 mL aliquots and sonicated until cells appeared to be visibly lysed. Sonicated products were centrifuged at 12 000 rcf and 4 °C for 15 min before purifying on a HisPur Ni-NTA column (Thermo Scientific) according to the manufacturer’s protocol. The eluted proteins were loaded into 10 kDa MWCO dialysis cassettes (Thermo Scientific) and dialyzed overnight in the storage buffer (50 mM Tris-HCl pH 7.5, 100 mM NaCl, 1 mM DTT, 1 mM EDTA, and 2% DMSO). Following dialysis, proteins were centrifuged at 12 000 rcf at 4 °C for 10 min to remove insoluble fractions. The supernatant was removed, and protein concentration was measured on a Nanodrop 2000 before subaliquoting and storage at −20 °C. Purification was verified by SDS-PAGE (Figure S9).

## Supporting Information Available

Supplementary Figures S1–S11: relative strengths of P_T7,strong_, P_T7,weak_, P_σ70,strong_, and P_σ70,weak_; protein expression curves for the experiment depicted in Figure 1; time-course analysis for the experiment depicted in Figure 1; assessment of plasmid crosstalk at the protein level in with basal levels of T7 RNAP; time-course analysis for the experiment depicted in Figure S4; assessment of plasmid crosstalk at the mRNA level in a different batch of T7 RNAP-enriched lysate; example data analysis of data depicted in Figure 2; repression of mRFP expression by expressed and purified LacI-sfGFP; SDS-PAGE gel for LacI-sfGFP; plasmid crosstalk in PUREfrex versus crude lysate; comparison of simulated (based on the txtlsim toolbox) and experimental data for crosstalk at the mRNA level; Supplementary Tables S1–S3: annotated sequences of all plasmids; concentrations of DNA or RNA templates used in all reported experiments; initial parameter values and search space lower and upper boundaries for the simulation results in Figure S11 (PDF)
