# Supplementary material for: Plasmid Crosstalk in Cell-Free Expression Systems
Source: ACS Synth Biol. 2023 Sep 27;12(10):2843–56. doi: 10.1021/acssynbio.3c00412 (PMC10594874; doi:10.1021/acssynbio.3c00412)
Supplement: Supplementary file 1 — sb3c00412_si_001.pdf [file sb3c00412_si_001.pdf]

Supporting Information for
Plasmid Crosstalk in Cell-Free Expression Systems

Fernanda Piorino*, Alexandra T. Patterson*, Yue Han, and Mark P. Styczynski**

* Contributed equally
** Corresponding author

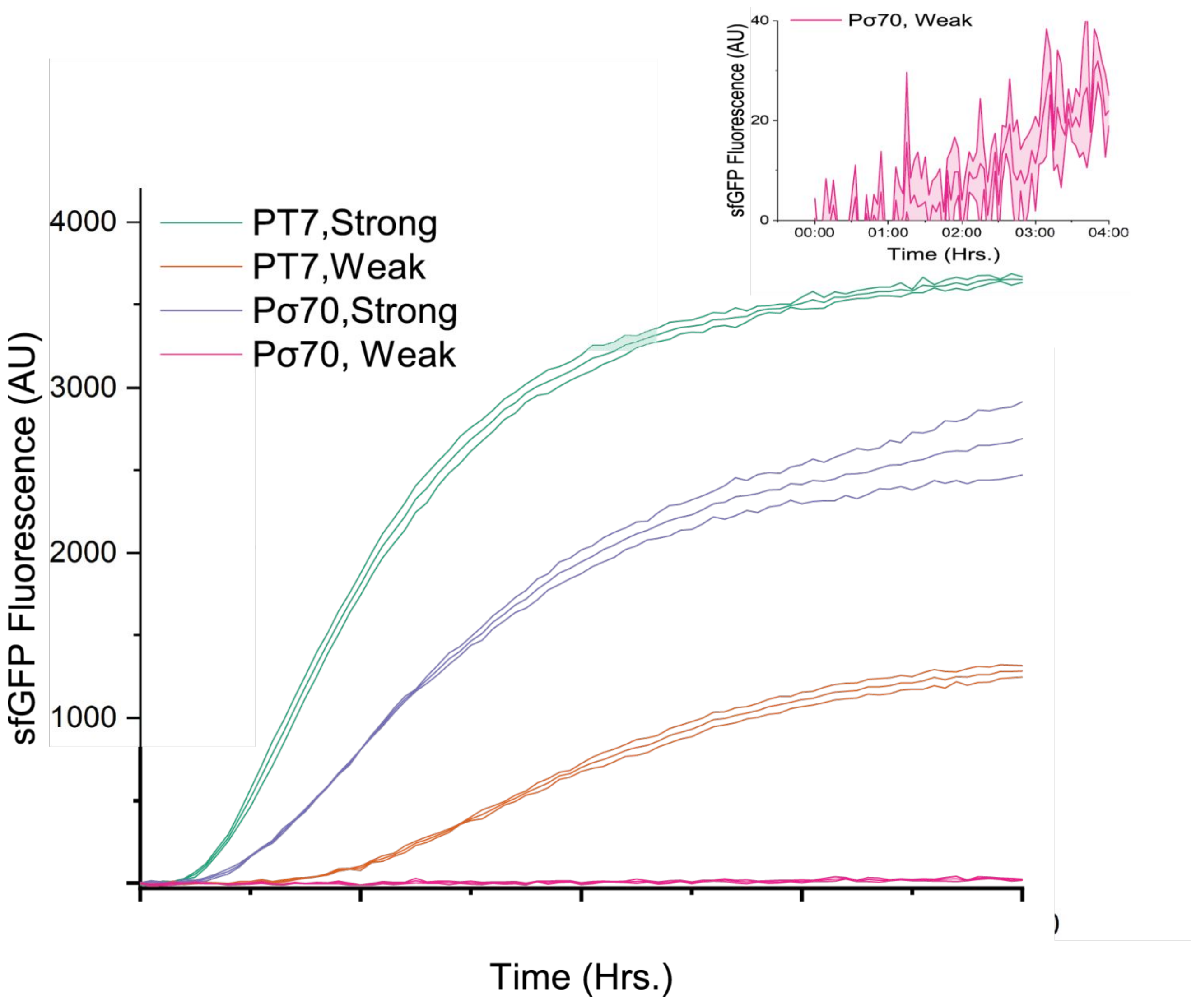

Figure S1. Relative strengths of $P_{T7,strong}$, $P_{T7,weak}$, $P_{\sigma70,strong}$, and $P_{\sigma70,weak}$. Reactions were run with 5 nM of plasmid expressing sfGFP from one of the four promoters. The inset provides a higher resolution view of the $P_{\sigma70,weak}$ output. Reactions were incubated at 37°C and background fluorescence was subtracted. The shaded region indicates the standard deviation of technical triplicates.

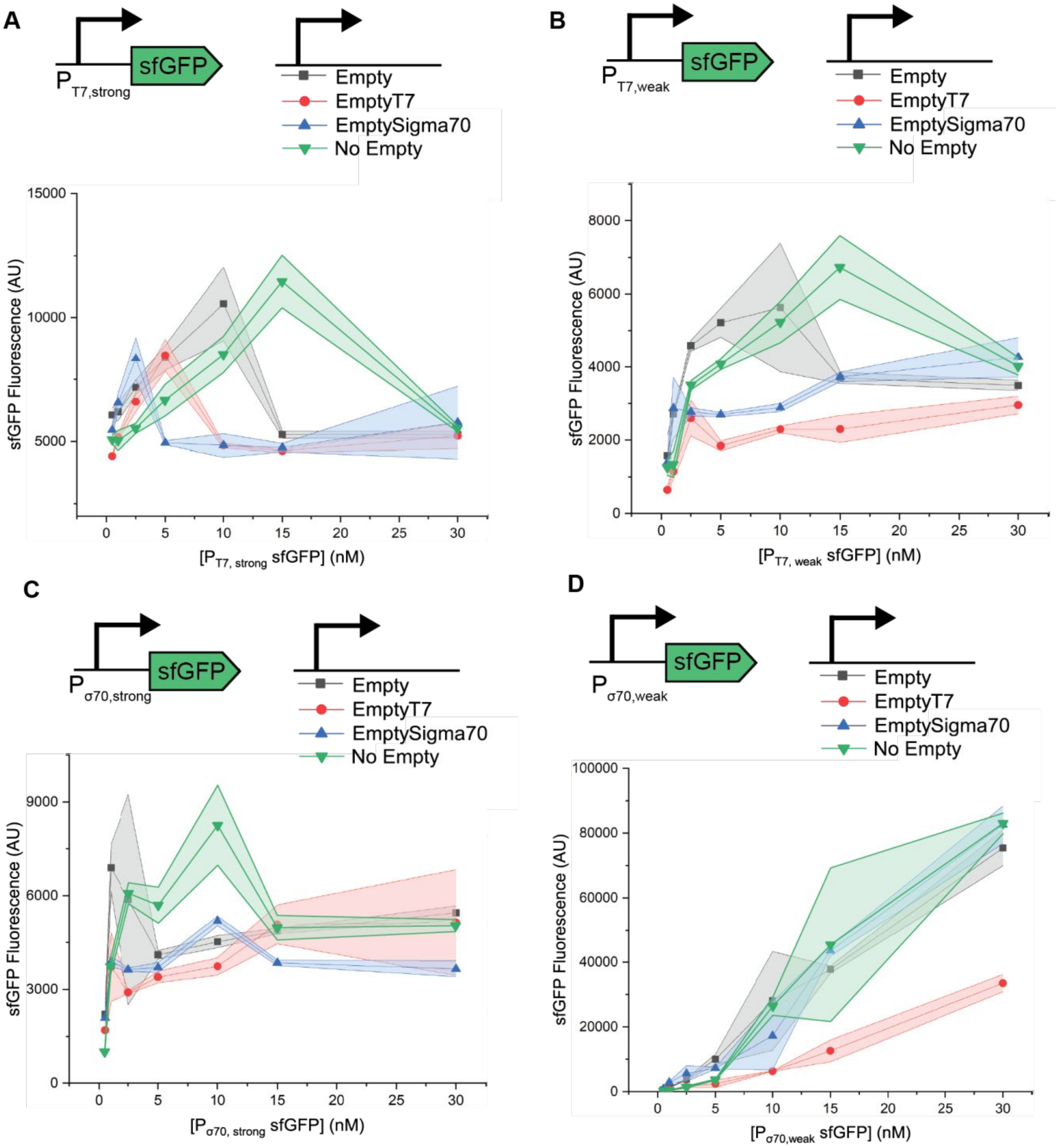

Figure S2. Protein expression curves corresponding to the data in Figure 1. GFP was transcribed from one of four promoters: (A) $P_{T7,strong}$, (B) $P_{T7,weak}$, (C) $P_{\sigma70,strong,}$ and (D) $P_{\sigma70,weak}$, each with three different types of empty vector and no additional plasmid added. In all subpanels, data were collected after a three-hour incubation at 37°C and background fluorescence was subtracted. The shaded region indicates the standard deviation of technical triplicates.

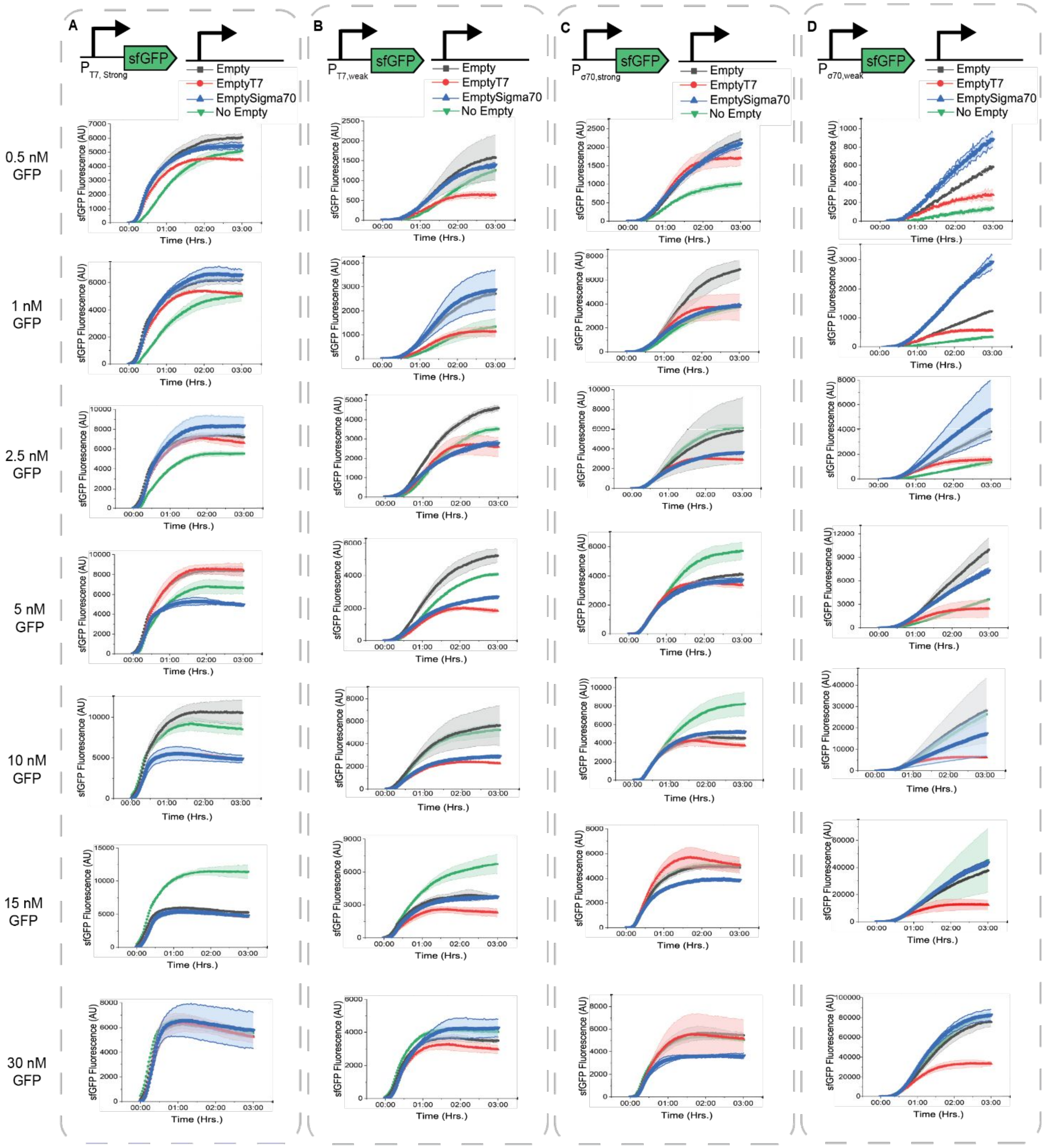

Figure S3. Protein expression time course data corresponding to the data in Figure 1. sfGFP was transcribed from one of four promoters: (A) $P_{T7,strong}$, (B) $P_{T7,weak}$, (C) $P_{\sigma70,strong}$, and (D) $P_{\sigma70,weak}$, each with three different types of empty vector and no additional plasmid added. In all subpanels, background fluorescence was subtracted. The shaded region indicates the standard deviation of technical triplicates.

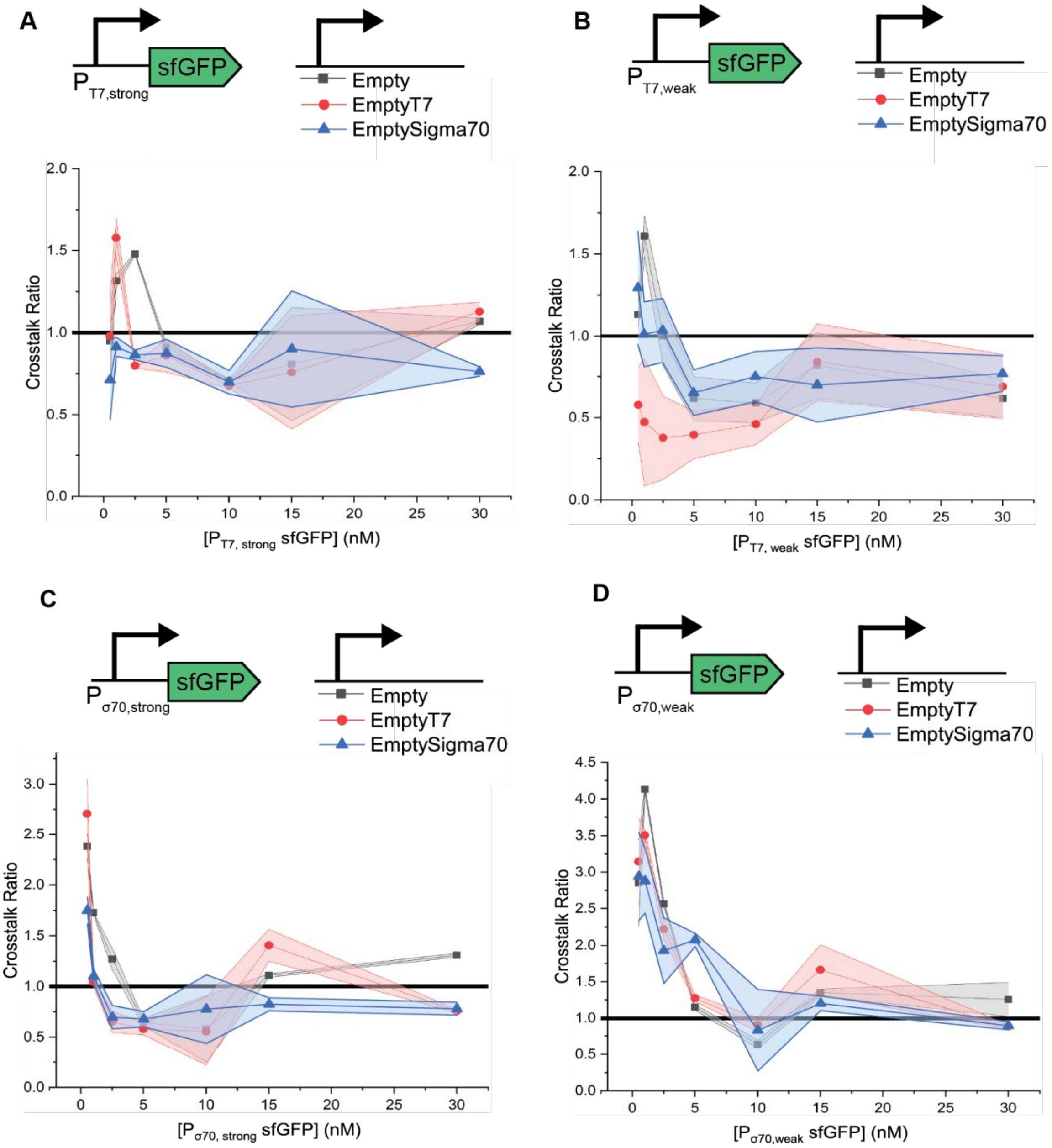

Figure S4. Assessment of plasmid crosstalk at the protein level in a lysate with basal T7 RNAP levels. The experimental design corresponds with that of the data in Figure 1. While the quantitative results for this lysate are not identical to those for the lysate used in Figure 1, most trends are consistent with a noteworthy exception of the results at low reporter concentrations for T7 reporters with EmptyT7 added. In all subpanels, data were collected after a three-hour incubation at 37°C and background fluorescence was subtracted. The shaded region indicates the standard deviation of technical triplicates.

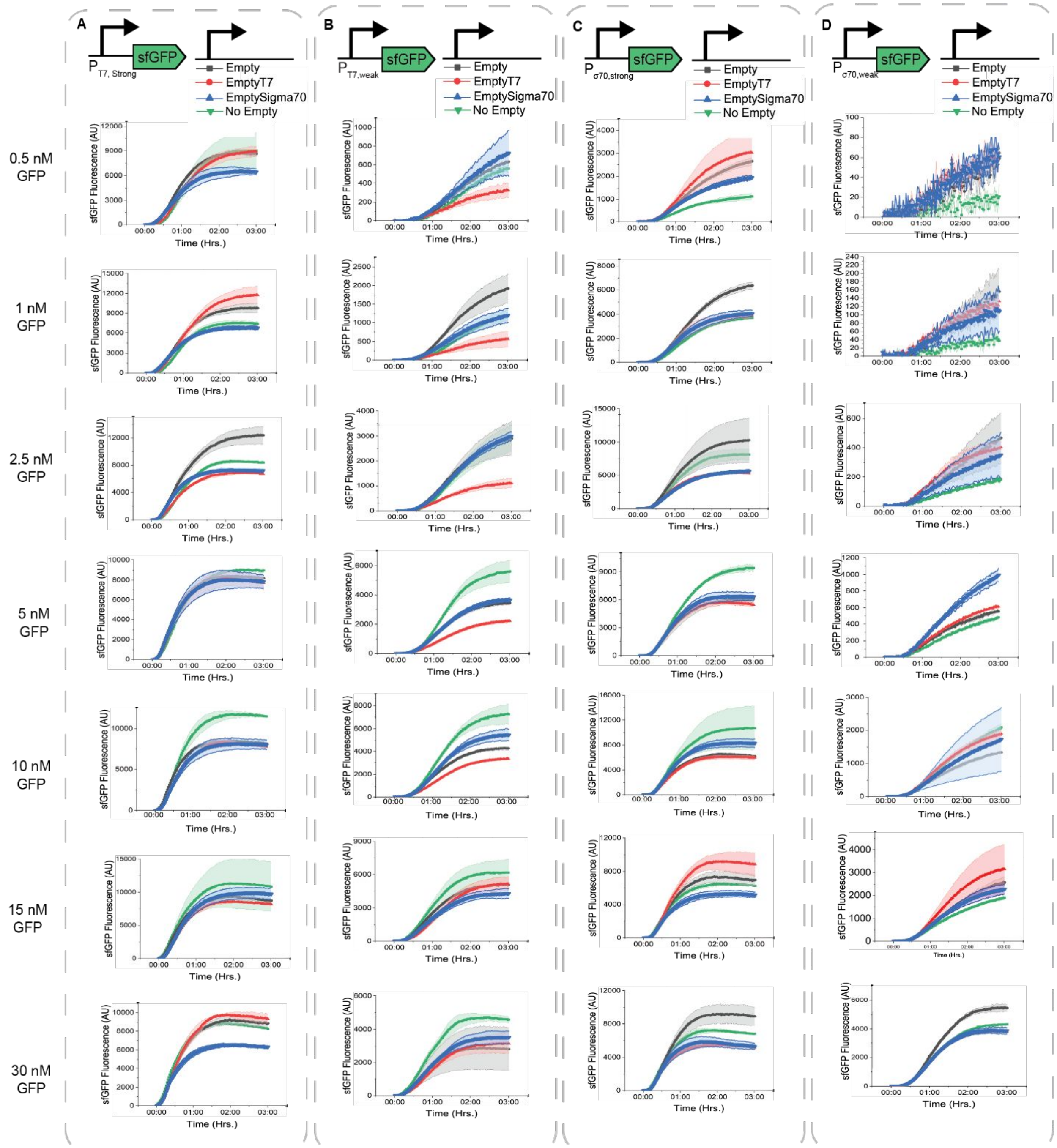

Figure S5. Protein expression time course data corresponding to the data in Figure S4. GFP was transcribed from one of four promoters: (A) $P_{T7,strong}$, (B) $P_{T7,weak}$, (C) $P_{\sigma70,strong}$, and (D) $P_{\sigma70,weak}$, each with three different types of empty vector and no additional plasmid added. In all subpanels, background fluorescence was subtracted. The shaded region indicates the standard deviation of technical triplicates.

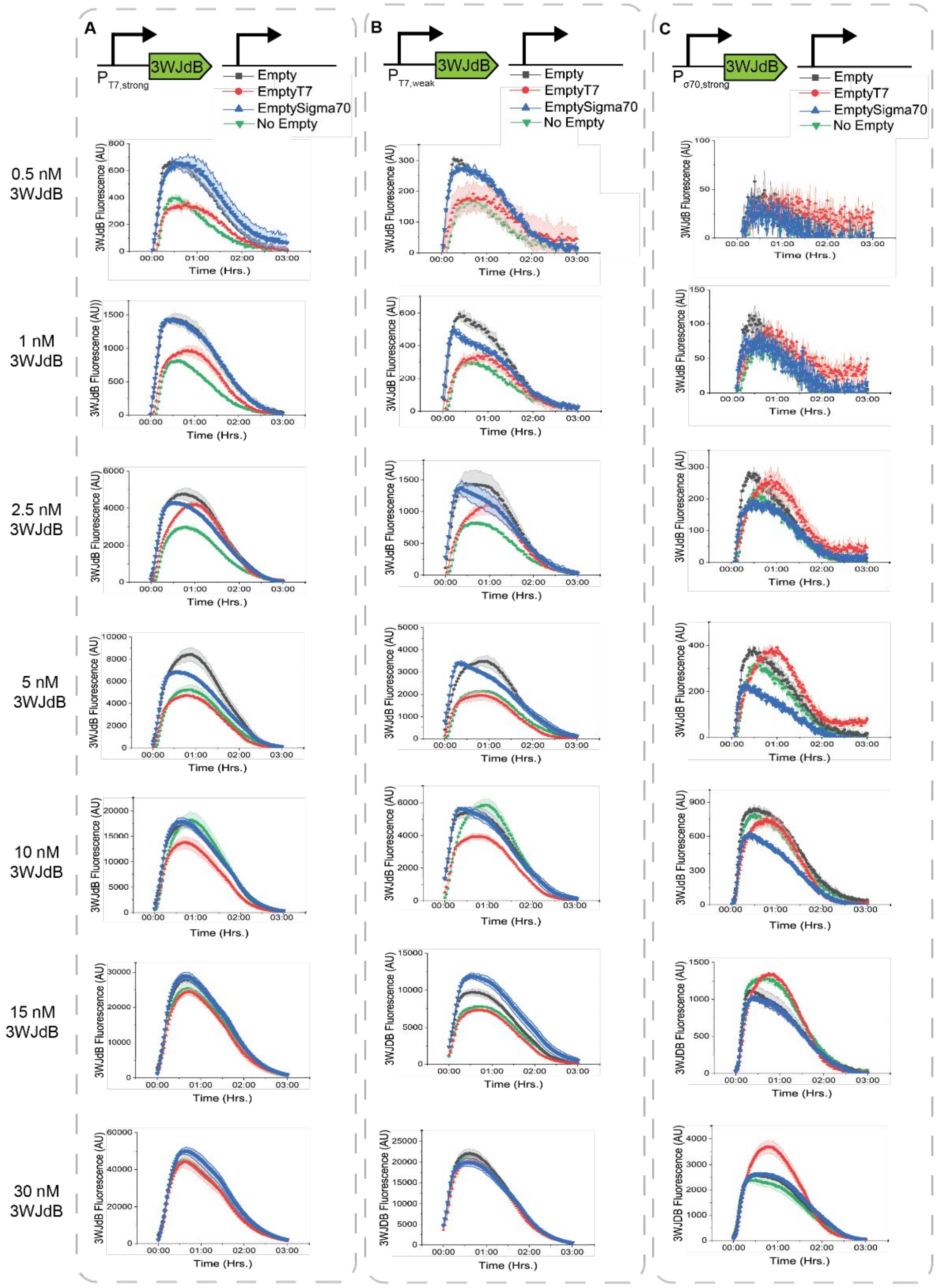

Figure S6. Assessment of plasmid crosstalk at the mRNA level. The same experimental design used for the data in Figure 2 was used, except the lysate was prepared by a different researcher than in Figure 2. All trends are qualitatively consistent, though quantitative values do not match exactly, as one would expect due to batch-to-batch and operator-level variability in cell-free lysate preparation. Data were processed as described in Figure S5. In all subpanels, reactions were run for 3 h at 37°C and background fluorescence was subtracted. The shaded region indicates the standard deviation of technical triplicates.

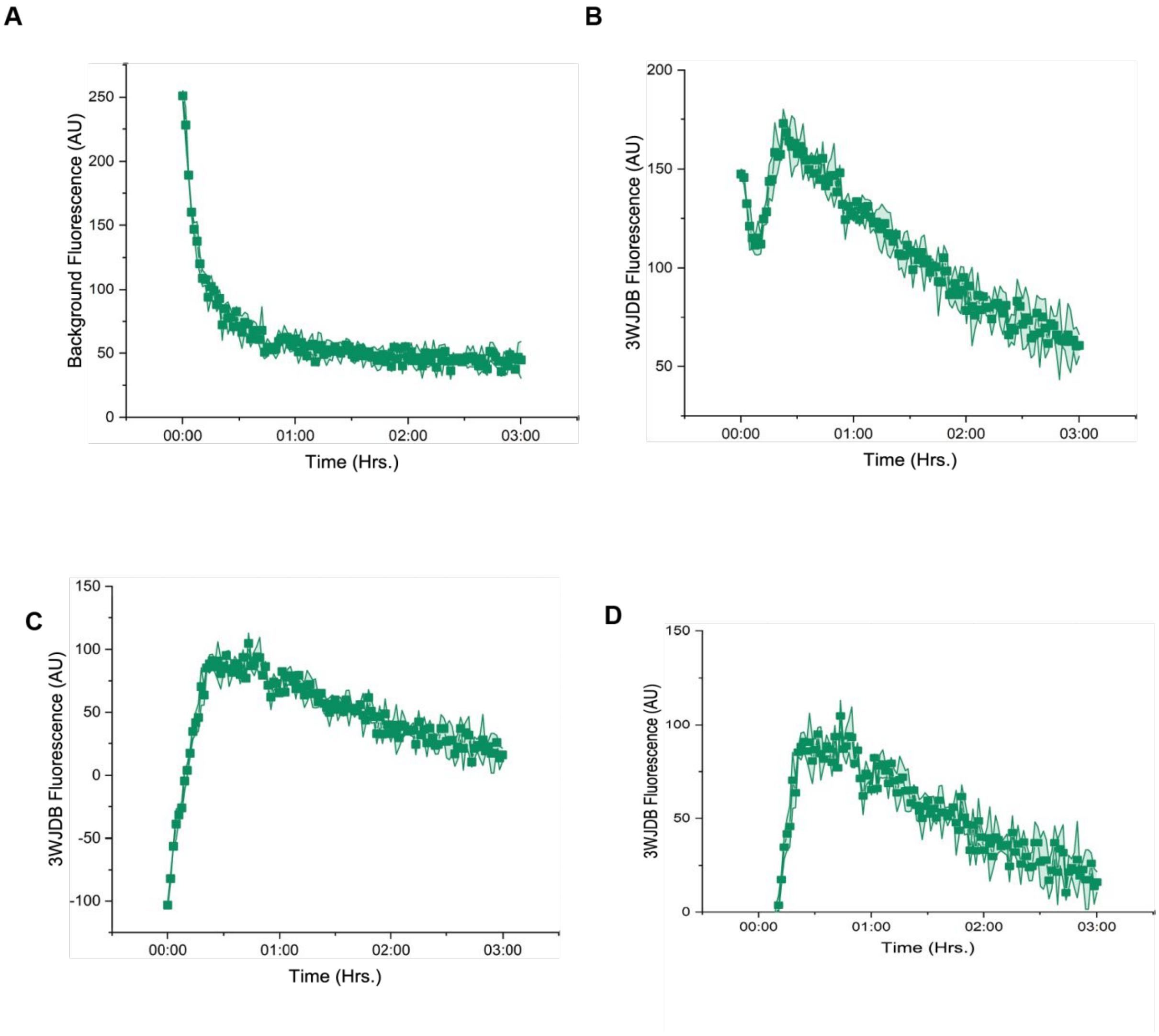

Figure S7. Example data analysis for data depicted in Figure 2 and Figure S6. (A) Background fluorescence of dye with no aptamer drops substantially as temperature in the plate reader equilibrates to 37°C after reaction assembly. (B) A representative 3JDB fluorescence curve with aptamer being expressed but without background subtraction also shows a drop early in the time course, though not as substantial. (C) A representative 3JDB fluorescence curve with background subtraction has negative fluorescence values at early time points due to the initial reduction in background fluorescence observed in panel A. (D) The final data as presented in Figure 2 are presented without negative fluorescence values since they are due to these artifacts. In all subpanels, reactions were run for 2 h at 37°C. The shaded region indicates the standard deviation of technical triplicates.

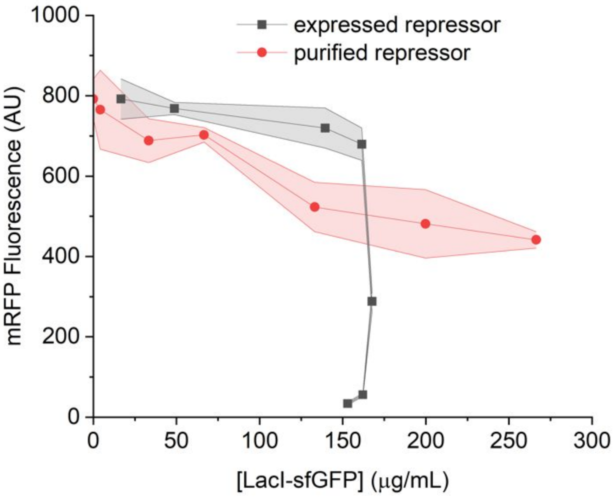

Figure S8. Repression of mRFP expression by expressed and purified LacI-sfGFP. RFP levels generally decrease with increasing concentration of purified LacI-sfGFP. At concentrations of repressor below ~150 μg/mL, the purified repressor enables stronger repression (compared to the fluorescence in the absence of repressor) of RFP levels than the expressed reporter. Above that threshold, however, increasing plasmid levels severely represses RFP expression, mediating much stronger repression than purified LacI. It is worth noting that the plasmid-expressed repressor curve does not seem to represent a function, with multiple fluorescence values corresponding to the same repressor concentration. This behavior arises from the fact that GFP fluorescence slightly decreases at high plasmid concentrations (as seen in Figure 5B), resulting in another (lower) fluorescence value being correlated to the same repressor concentration.

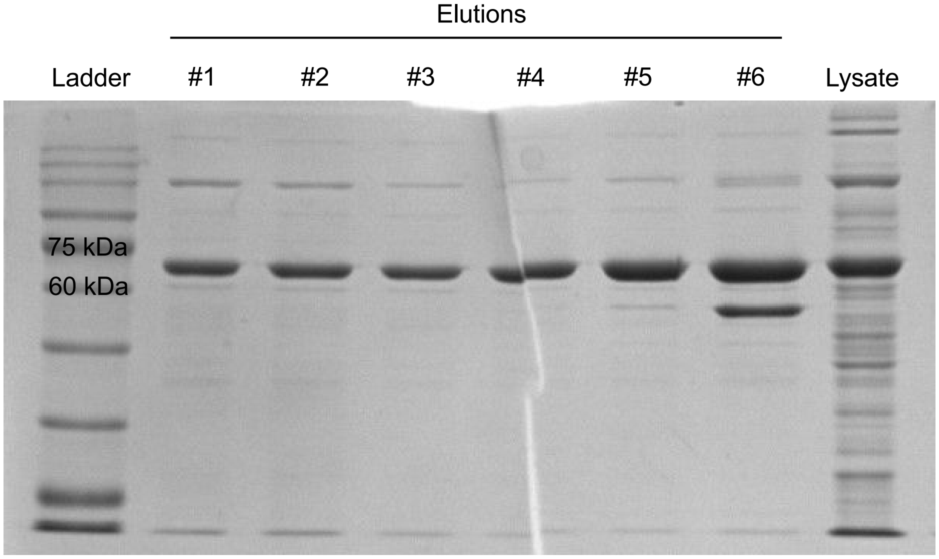

Figure S9. SDS-PAGE gel for LacI-sfGFP. The size of the LacI-sfGFP fusion is approximately 65 kDa. The protein ladder is a Trident Prestained (GTX50875). Elution #4 was used in the experiments described in Figure 5 and Figure S8.

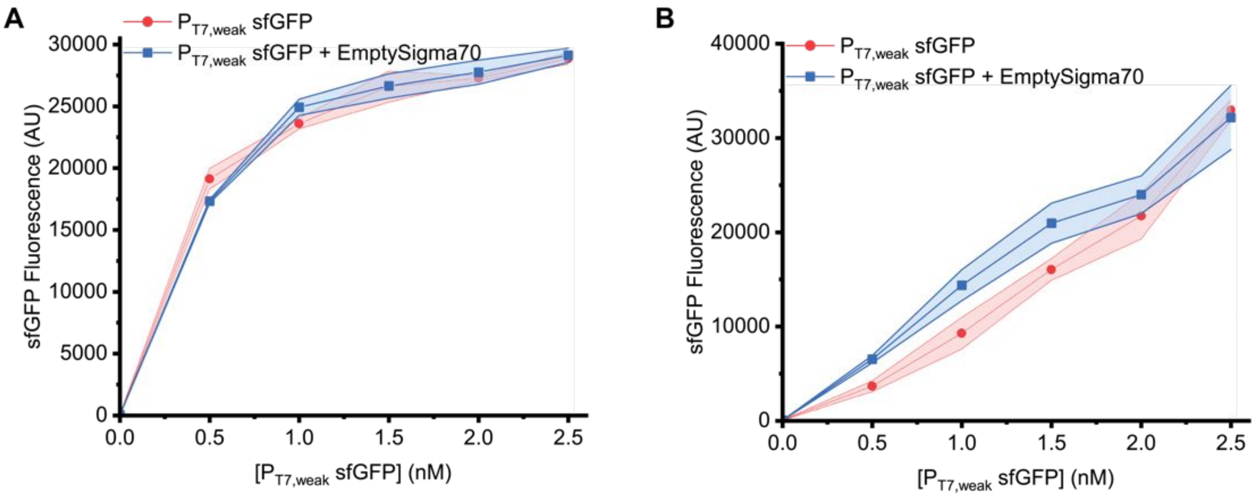

Figure S10. Plasmid crosstalk in PUREfrex versus crude lysate. (A) In PUREfrex, minimal crosstalk is observed, as addition of 10 nM EmptySigma70 does not substantially alter sfGFP levels. (B) In a T7 RNAP-enriched crude lysate, EmptySigma70 induces positive crosstalk at low concentrations of the sfGFP plasmid. In all subpanels, data were collected after a three-hour incubation at 37°C. The shaded region indicates the standard deviation of technical triplicates.

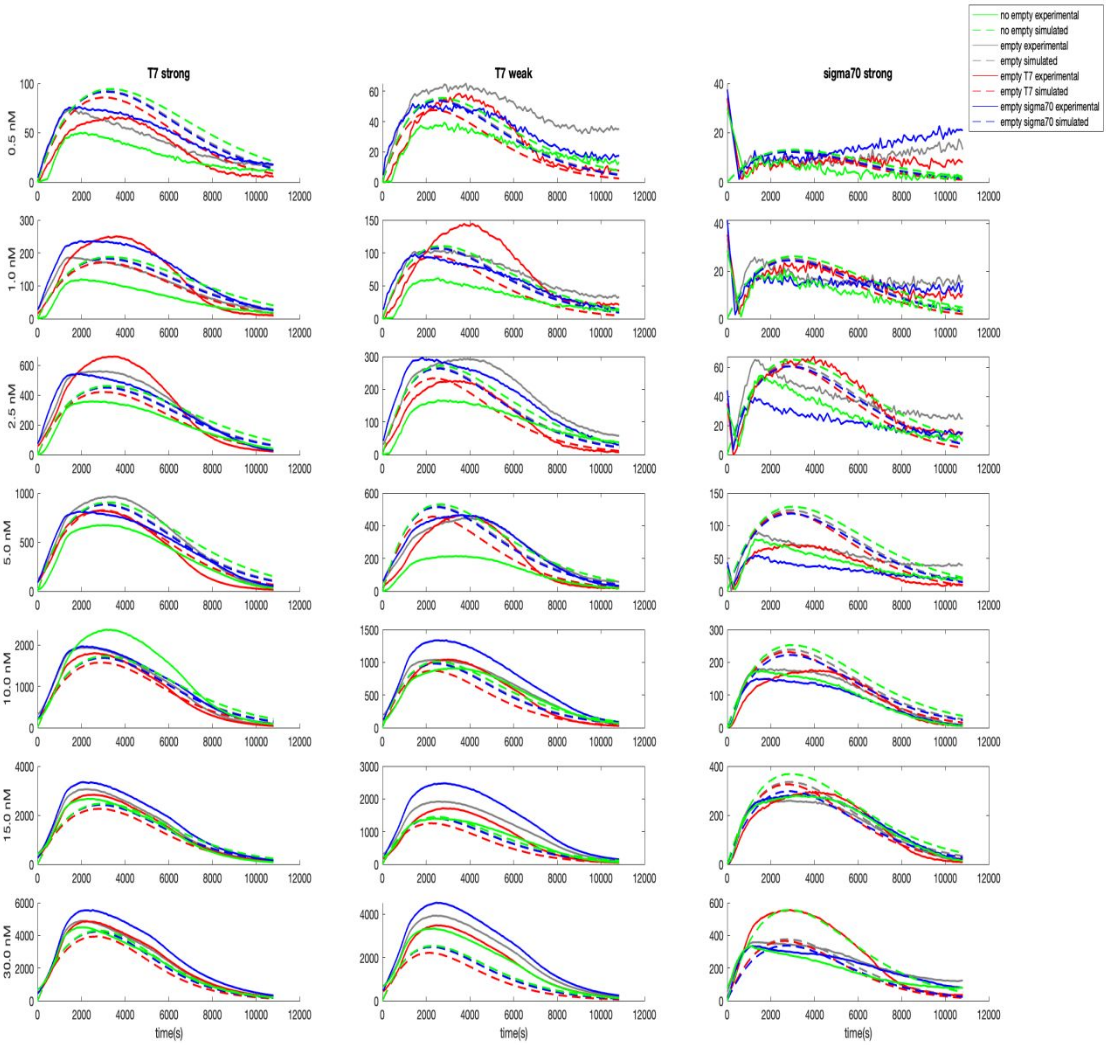

Figure S11. Comparison of simulated (based on the txtlsim toolbox, dotted curves) and experimental (solid curves) data for crosstalk at the mRNA level. The experimental data are reproduced from Figure 2. Green represents the "no empty" condition, such that a green curve below other colors indicates positive crosstalk. Positive crosstalk is observed across many conditions in the experimental data but could not be recapitulated in the model.

Table S1. Description of plasmid parts and DNA sequences in this paper.

$P_{T7,strong}$ sfGFP	Plasmid encoding sfGFP expression under $P_{T7,strong}$
$P_{T7,strong}$ Stability hairpin RBS sfGFP Terminator Kanamycin resistance cassette ColE1 origin<br><br>agatcaaaggatcttcttgagatcctttttttctgcgcgtaatctgctgcttgcaaacaaaaaaaccaccgctaccagcggtggtttgtttgcc<br>ggatcaagagctaccaactctttttccgaaggtaactggcttcagcagagcgcagataccaaatactgttcttctagtgtagccgtagttag<br>gccaccacttcaagaactctgtagcaccgcctacatacctcgctctgctaatcctgttaccagtggctgctgccagtggcgataagtcgtg<br>tcttaccgggttggactcaagacgatagttaccggataaggcgcagcggtcgggctgaacggggggttcgtgcacacagcccagcttg<br>gagcgaacgacctacaccgaactgagatacctacagcgtgagctatgagaaagcgccacgcttcccgaagggagaaaggcggaca<br>ggtatccggtaagcggcagggtcggaacaggagagcgcacgagggagcttccagggggaaacgcctggtatctttatagtcctgtcg<br>ggtttcgccacctctgacttgagcgtcgatttttgtgatgctcgtcaggggggcggagcctatggaaaaacgccagcaacgcgatcccg<br>cgaaattaatacgactcactatagggagaccacaacggtttccctctagaaataattttgtttaactttaagaaggagatatacatATGA<br>GCAAAGGTGAAGAACTGTTTACCGGCGTTGTGCCGATTCTGGTGGAACTGGATGG<br>CGATGTGAACGGTCACAAATTCAGCGTGCGTGGTGAAGGTGAAGGCGATGCCACG<br>ATTGGCAAACTGACGCTGAAATTTATCTGCACCACCGGCAAACTGCCGGTGCCGT<br>GGCCGACGCTGGTGACCACCCTGACCTATGGCGTTCAGTGTTTTAGTCGCTATCCG<br>GATCACATGAAACGTCACGATTTCTTTAAATCTGCAATGCCGGAAGGCTATGTGC<br>AGGAACGTACGATTAGCTTTAAAGATGATGGCAAATATAAAACGCGCGCCGTTGT<br>GAAATTTGAAGGCGATACCCTGGTGAACCGCATTGAACTGAAAGGCACGGATTTT<br>AAAGAAGATGGCAATATCCTGGGCCATAAACTGGAATACAACTTTAATAGCCATA<br>ATGTTTATATTACGGCGGATAAACAGAAAAATGGCATCAAAGCGAATTTTACCGT<br>TCGCCATAACGTTGAAGATGGCAGTGTGCAGCTGGCAGATCATTATCAGCAGAAT<br>ACCCCGATTGGTGATGGTCCGGTGCTGCTGCCGGATAATCATTATCTGAGCACGCA<br>GACCGTTCTGTCTAAAGATCCGAACGAAAAAGGCACGCGGGACCACATGGTTCTG<br>CACGAATATGTGAATGCGGCAGGTATTACGTGGAGCCATCCGCAGTTCGAAAAAT<br>AAgtcgaccggctgctaacaaagcccgaaaggaagctgagttggctgctgccaccgctgagcaataactagcataaccccttgggg<br>cctctaaacgggtcttgaggggttttttgctgaaagccaattctgattagaaaaactcatcgagcatcaaatgaaactgcaatttattcatatc<br>aggattatcaataccatatttttgaaaaagccgtttctgtaatgaaggagaaaactcaccgaggcagttccataggatggcaagatcctggt<br>atcggtctgcgattccgactcgtccaacatcaatacaacctattaatttcccctcgtcaaaaataaggttatcaagtgagaaatcaccatgag<br>tgacgactgaatccggtgagaatggcaaaagcttatgcatttctttccagacttgttcaacaggccagccattacgctcgtcatcaaaatca<br>ctcgcatcaaccaaaccgttattcattcgtgattgcgcctgagcgagacgaaatacgcgatcgctgttaaaaggacaattacaaacagga<br>atcgaatgcaaccggcgcaggaacactgccagcgcatcaacaatattttcacctgaatcaggatattcttctaatacctggaatgctgttttc<br>ccggggatcgcagtggtgagtaaccatgcatcatcaggagtacggataaaatgcttgatggtcggaagaggcataaattccgtcagcca<br>gtttagtctgaccatctcatctgtaacatcattggcaacgctacctttgccatgtttcagaaacaactctggcgcatcgggcttcccatacaat<br>cgatagattgtcgcacctgattgcccgacattatcgcgagcccatttatacccatataaatcagcatccatgttggaatttaatcgcggcttc<br>gagcaagacgtttcccgttgaatatggctcataacacccttgtattactgtttatgtaagcagacagttttattgttcatgatgatatatttttat<br>cttgtgcaatgtaacatcagagattttgagacacaacgtg	

$P_{T7,weak}$ sfGFP	Plasmid encoding sfGFP expression under $P_{T7,weak}$
$P_{T7,weak}$ Stability hairpin RBS sfGFP Terminator Kanamycin resistance cassette ColE1 origin<br><br>agatcaaaggatcttcttgagatcctttttttctgcgcgtaatctgctgcttgcaaacaaaaaaaccaccgctaccagcggtggtttgtttgcc<br>ggatcaagagctaccaactctttttccgaaggtaactggcttcagcagagcgcagataccaaatactgttcttctagtgtagccgtagttag<br>gccaccacttcaagaactctgtagcaccgcctacatacctcgctctgctaatcctgttaccagtggctgctgccagtggcgataagtcgtg<br>tcttaccgggttggactcaagacgatagttaccggataaggcgcagcggtcgggctgaacggggggttcgtgcacacagcccagcttg<br>gagcgaacgacctacaccgaactgagatacctacagcgtgagctatgagaaagcgccacgcttcccgaagggagaaaggcggaca	

ggtatccggtaagcggcagggtcggaacaggagagcgcacgagggagcttccagggggaaacgcctggtatctttatagtcctgtcg
ggtttcgccacctctgacttgagcgtcgatttttgtgatgctcgtcaggggggcggagcctatggaaaaacgccagcaacgcgatcccg
cgaaatTAATACGACTCACTAAAGGgagaccacaacggtttccctctagaaataattttgtttaactttaagaaggagat
atacatATGAGCAAAGGTGAAGAACTGTTTACCGGCGTTGTGCCGATTCTGGTGGAAC
TGGATGGCGATGTGAACGGTCACAAATTCAGCGTGCGTGGTGAAGGTGAAGGCGA
TGCCACGATTGGCAAACTGACGCTGAAATTTATCTGCACCACCGGCAAACTGCCG
GTGCCGTGGCCGACGCTGGTGACCACCCTGACCTATGGCGTTCAGTGTTTTAGTCG
CTATCCGGATCACATGAAACGTCACGATTTCTTTAAATCTGCAATGCCGGAAGGCT
ATGTGCAGGAACGTACGATTAGCTTTAAAGATGATGGCAAATATAAAACGCGCGC
CGTTGTGAAATTTGAAGGCGATACCCTGGTGAACCGCATTGAACTGAAAGGCACG
GATTTTAAAGAAGATGGCAATATCCTGGGCCATAAACTGGAATACAACTTTAATA
GCCATAATGTTTATATTACGGCGGATAAACAGAAAAATGGCATCAAAGCGAATTT
TACCGTTCGCCATAACGTTGAAGATGGCAGTGTGCAGCTGGCAGATCATTATCAG
CAGAATACCCCGATTGGTGATGGTCCGGTGCTGCTGCCGGATAATCATTATCTGAG
CACGCAGACCGTTCTGTCTAAAGATCCGAACGAAAAAGGCACGCGGGACCACATG
GTTCTGCACGAATATGTGAATGCGGCAGGTATTACGTGGAGCCATCCGCAGTTCG
AAAAATAAgtcgaccggctgctaacaaagcccgaaaggaagctgagttggctgctgccaccgctgagcaataactagcataac
cccttggggcctctaaacgggtcttgaggggttttttgctgaaagccaattctgattagaaaaactcatcgagcatcaaatgaaactgcaat
ttattcatatcaggattatcaataccatatttttgaaaaagccgtttctgtaatgaaggagaaaactcaccgaggcagttccataggatggca
agatcctggtatcggtctgcgattccgactcgtccaacatcaatacaacctattaatttcccctcgtcaaaaataaggttatcaagtgagaaa
tcaccatgagtgacgactgaatccggtgagaatggcaaaagcttatgcatttctttccagacttgttcaacaggccagccattacgctcgtc
atcaaaatcactcgcatcaaccaaaccgttattcattcgtgattgcgcctgagcgagacgaaatacgcgatcgctgttaaaaggacaatta
caaacaggaatcgaatgcaaccggcgcaggaacactgccagcgcatcaacaatattttcacctgaatcaggatattcttctaatacctgg
aatgctgttttcccggggatcgcagtggtgagtaaccatgcatcatcaggagtacggataaaatgcttgatggtcggaagaggcataaatt
ccgtcagccagtttagtctgaccatctcatctgtaacatcattggcaacgctacctttgccatgtttcagaaacaactctggcgcatcgggct
tcccatacaatcgatagattgtcgcacctgattgcccgacattatcgcgagcccatttatacccatataaatcagcatccatgttggaatttaa
tcgcggcttcgagcaagacgtttcccgttgaatatggctcataacaccccttgtattactgtttatgtaagcagacagttttattgttcatgatg
atatatttttatcttgtgcaatgtaacatcagagattttgagacacaacgtg

P $_{\sigma 70,strong}$ sfGFP	Plasmid encoding sfGFP expression under P $_{\sigma 70,strong}$

P $_{\sigma 70,strong}$ Stability hairpin RBS sfGFP Terminator Kanamycin resistance cassette ColE1 origin

agatcaaaggatcttcttgagatcctttttttctgcgcgtaatctgctgcttgcaaacaaaaaaaccaccgctaccagcggtggtttgtttgcc
ggatcaagagctaccaactctttttccgaaggtaactggcttcagcagagcgcagataccaaatactgttcttctagtgtagccgtagttag
gccaccacttcaagaactctgtagcaccgcctacatacctcgctctgctaatcctgttaccagtggctgctgccagtggcgataagtcgtg
tcttaccgggttggactcaagacgatagttaccggataaggcgcagcggtcgggctgaacggggggttcgtgcacacagcccagcttg
gagcgaacgacctacaccgaactgagatacctacagcgtgagctatgagaaagcgccacgcttcccgaagggagaaaggcggaca
ggtatccggtaagcggcagggtcggaacaggagagcgcacgagggagcttccagggggaaacgcctggtatctttatagtcctgtcg
ggtttcgccacctctgacttgagcgtcgatttttgtgatgctcgtcaggggggcggagcctatggaaaaacgccagcaacgcgatcccg
cgaaatttgacagctagctcagtcctaggtataatactagtccacaacggtttccctctagaaataattttgtttaactttaagaaggagatata
catATGAGCAAAGGTGAAGAACTGTTTACCGGCGTTGTGCCGATTCTGGTGGAACT
GGATGGCGATGTGAACGGTCACAAATTCAGCGTGCGTGGTGAAGGTGAAGGCGAT
GCCACGATTGGCAAACTGACGCTGAAATTTATCTGCACCACCGGCAAACTGCCGG
TGCCGTGGCCGACGCTGGTGACCACCCTGACCTATGGCGTTCAGTGTTTTAGTCGC
TATCCGGATCACATGAAACGTCACGATTTCTTTAAATCTGCAATGCCGGAAGGCTA
TGTGCAGGAACGTACGATTAGCTTTAAAGATGATGGCAAATATAAAACGCGCGCC

GTTGTGAAATTTGAAGGCGATACCCTGGTGAACCGCATTGAACTGAAAGGCACGG
ATTTTAAAGAAGATGGCAATATCCTGGGCCATAAACTGGAATACAACTTTAATAG
CCATAATGTTTATATTACGGCGGATAAACAGAAAAATGGCATCAAAGCGAATTTT
ACCGTTCGCCATAACGTTGAAGATGGCAGTGTGCAGCTGGCAGATCATTATCAGC
AGAATACCCCGATTGGTGATGGTCCGGTGCTGCTGCCGGATAATCATTATCTGAGC
ACGCAGACCGTTCTGTCTAAAGATCCGAACGAAAAAGGCACGCGGGACCACATG
GTTCTGCACGAATATGTGAATGCGGCAGGTATTACGTGGAGCCATCCGCAGTTCG
AAAAATAAgtcgaccggctgctaacaaagcccgaaaggaagctgagttggctgctgccagaagcttgggcccgaacaaaaac
tcatctcagaagaggatctgaatagcgccgtcgaccatcatcatcatcatcattgagtttaaacggtctccagcttggctgttttggcggatg
agagaagattttcagcctgatacagattaaatcagaacgcagaagcggtctgataaaacagaatttgcctggcggcagtagcgcggtgg
tcccacctgaccccatgccgaactcagaagtgaaacgccgtagcgccgatggtagtgtggggtctccccatgcgagagtagggaactg
ccaggcatcaaataaaacgaaaggctcagtcgaaagactgggcctttcgttttatctgttgtttgtcggtgaactctgaaagccaattctgat
tagaaaaactcatcgagcatcaaatgaaactgcaatttattcatatcaggattatcaataccatatttttgaaaaagccgtttctgtaatgaag
gagaaaactcaccgaggcagttccataggatggcaagatcctggtatcggtctgcgattccgactcgtccaacatcaatacaacctatta
atttcccctcgtcaaaaataaggttatcaagtgagaaatcaccatgagtgacgactgaatccggtgagaatggcaaaagcttatgcatttct
ttccagacttgttcaacaggccagccattacgctcgtcatcaaaatcactcgcatcaaccaaaccgttattcattcgtgattgcgcctgagc
gagacgaaatacgcgatcgctgttaaaaggacaattacaaacaggaatcgaatgcaaccggcgcaggaacactgccagcgcatcaac
aatattttcacctgaatcaggatattcttctaatacctggaatgctgttttcccggggatcgcagtggtgagtaaccatgcatcatcaggagta
cggataaaatgcttgatggtcggaagaggcataaattccgtcagccagtttagtctgaccatctcatctgtaacatcattggcaacgctacc
tttgccatgtttcagaaacaactctggcgcatcgggcttcccatacaatcgatagattgtcgcacctgattgcccgacattatcgcgagccc
atttatacccatataaatcagcatccatgttggaatttaatcgcggcttcgagcaagacgtttcccgttgaatatggctcataacaccccttgt
attactgtttatgtaagcagacagttttattgttcatgatgatatatttttatcttgtgcaatgtaacatcagagattttgagacacaacgtg

$P_{\sigma70,weak\ sfGFP}$	Plasmid encoding sfGFP expression under $P_{\sigma70,weak}$

$P_{\sigma70,weak}$ Stability hairpin RBS sfGFP Terminator Kanamycin resistance cassette ColE1 origin

agatcaaaggatcttcttgagatcctttttttctgcgcgtaatctgctgcttgcaaacaaaaaaaccaccgctaccagcggtggtttgtttgcc
ggatcaagagctaccaactctttttccgaaggtaactggcttcagcagagcgcagataccaaatactgttcttctagtgtagccgtagttag
gccaccacttcaagaactctgtagcaccgcctacatacctcgctctgctaatcctgttaccagtggctgctgccagtggcgataagtcgtg
tcttaccgggttggactcaagacgatagttaccggataaggcgcagcggtcgggctgaacggggggttcgtgcacacagcccagcttg
gagcgaacgacctacaccgaactgagatacctacagcgtgagctatgagaaagcgccacgcttcccgaagggagaaaggcggaca
ggtatccggtaagcggcagggtcggaacaggagagcgcacgagggagcttccagggggaaacgcctggtatctttatagtcctgtcg
ggtttcgccacctctgacttgagcgtcgatttttgtgatgctcgtcaggggggcggagcctatggaaaaacgccagcaacgcgatcccg
cgaaatttacggctagctcagtcctaggtactatgctagcccacaacggtttccctctagaaataattttgtttaactttaagaaggagatat
acatATGAGCAAAGGTGAAGAACTGTTTACCGGCGTTGTGCCGATTCTGGTGGAACT
GGATGGCGATGTGAACGGTCACAAATTCAGCGTGCGTGGTGAAGGTGAAGGCGAT
GCCACGATTGGCAAACTGACGCTGAAATTTATCTGCACCACCGGCAAACTGCCGG
TGCCGTGGCCGACGCTGGTGACCACCCTGACCTATGGCGTTCAGTGTTTTAGTCGC
TATCCGGATCACATGAAACGTCACGATTTCTTTAAATCTGCAATGCCGGAAGGCTA
TGTGCAGGAACGTACGATTAGCTTTAAAGATGATGGCAAATATAAAACGCGCGCC
GTTGTGAAATTTGAAGGCGATACCCTGGTGAACCGCATTGAACTGAAAGGCACGG
ATTTTAAAGAAGATGGCAATATCCTGGGCCATAAACTGGAATACAACTTTAATAG
CCATAATGTTTATATTACGGCGGATAAACAGAAAAATGGCATCAAAGCGAATTTT
ACCGTTCGCCATAACGTTGAAGATGGCAGTGTGCAGCTGGCAGATCATTATCAGC
AGAATACCCCGATTGGTGATGGTCCGGTGCTGCTGCCGGATAATCATTATCTGAGC
ACGCAGACCGTTCTGTCTAAAGATCCGAACGAAAAAGGCACGCGGGACCACATG

GTTCTGCACGAATATGTGAATGCGGCAGGTATTACGTGGAGCCATCCGCAGTTCG
AAAAATAAgtcgaccggctgctaacaaagcccgaaaggaagctgagttggctgctgccagaagcttgggcccgaacaaaaac
tcatctcagaagaggatctgaatagcgccgtcgaccatcatcatcatcatcattgagtttaaacggtctccagcttggctgttttggcggatg
agagaagattttcagcctgatacagattaaatcagaacgcagaagcggtctgataaaacagaatttgcctggcggcagtagcgcggtgg
tcccacctgaccccatgccgaactcagaagtgaaacgccgtagcgccgatggtagtgtggggtctccccatgcgagagtagggaactg
ccaggcatcaaataaaacgaaaggctcagtcgaaagactgggcctttcgttttatctgttgtttgtcggtgaactctgaaagccaattctgat
tagaaaaactcatcgagcatcaaatgaaactgcaatttattcatatcaggattatcaataccatatttttgaaaaagccgtttctgtaatgaag
gagaaaactcaccgaggcagttccataggatggcaagatcctggtatcggtctgcgattccgactcgtccaacatcaatacaacctatta
atttcccctcgtcaaaaataaggttatcaagtgagaaatcaccatgagtgacgactgaatccggtgagaatggcaaaagcttatgcatttct
ttccagacttgttcaacaggccagccattacgctcgtcatcaaaatcactcgcatcaaccaaaccgttattcattcgtgattgcgcctgagc
gagacgaaatacgcgatcgctgttaaaaggacaattacaaacaggaatcgaatgcaaccggcgcaggaacactgccagcgcatcaac
aatattttcacctgaatcaggatattcttctaatacctggaatgctgttttcccggggatcgcagtggtgagtaaccatgcatcatcaggagta
cggataaaatgcttgatggtcggaagaggcataaattccgtcagccagtttagtctgaccatctcatctgtaacatcattggcaacgctacc
tttgccatgtttcagaaacaactctggcgcatcgggcttcccatacaatcgatagattgtcgcacctgattgcccgacattatcgcgagccc
atttatacccatataaatcagcatccatgttggaatttaatcgcggcttcgagcaagacgtttcccgttgaatatggctcataacacccccttgt
attactgtttatgtaagcagacagttttattgttcatgatgatatatttttatcttgtgcaatgtaacatcagagattttgagacacaacgtg

Empty	Empty vector only encoding an antibiotic resistance and origin of replication

Kanamycin resistance cassette ColE1 origin

agatcaaaggatcttcttgagatcctttttttctgcgcgtaatctgctgcttgcaaacaaaaaaaccaccgctaccagcggtggtttgtttgcc
ggatcaagagctaccaactctttttccgaaggtaactggcttcagcagagcgcagataccaaatactgttcttctagtgtagccgtagttag
gccaccacttcaagaactctgtagcaccgcctacatacctcgctctgctaatcctgttaccagtggctgctgccagtggcgataagtcgtg
tcttaccgggttggactcaagacgatagttaccggataaggcgcagcggtcgggctgaacggggggttcgtgcacacagcccagcttg
gagcgaacgacctacaccgaactgagatacctacagcgtgagctatgagaaagcgccacgcttcccgaagggagaaaggcggaca
ggtatccggtaagcggcagggtcggaacaggagagcgcacgagggagcttccagggggaaacgcctggtatctttatagtcctgtcg
ggtttcgccacctctgacttgagcgtcgatttttgtgatgctcgtcaggggggcggagcctatggaaaaacgccagcaacgcgatcccg
cgaaattaatacgactcactatagggagaacaaagcccgaaaggaagctgagttggctgctgccaccgctgagcaataac
tagcataaccccttggggcctctaaacgggtcttgaggggttttttgctgaaagccaattctgattagaaaaactcatcgagcatcaaatga
aactgcaatttattcatatcaggattatcaataccatatttttgaaaaagccgtttctgtaatgaaggagaaaactcaccgaggcagttccata
ggatggcaagatcctggtatcggtctgcgattccgactcgtccaacatcaatacaacctattaatttcccctcgtcaaaaataaggttatcaa
gtgagaaatcaccatgagtgacgactgaatccggtgagaatggcaaaagcttatgcatttctttccagacttgttcaacaggccagccatt
acgctcgtcatcaaaatcactcgcatcaaccaaaccgttattcattcgtgattgcgcctgagcgagacgaaatacgcgatcgctgttaaaa
ggacaattacaaacaggaatcgaatgcaaccggcgcaggaacactgccagcgcatcaacaatattttcacctgaatcaggatattcttct
aatacctggaatgctgttttcccggggatcgcagtggtgagtaaccatgcatcatcaggagtacggataaaatgcttgatggtcggaaga
ggcataaattccgtcagccagtttagtctgaccatctcatctgtaacatcattggcaacgctacctttgccatgtttcagaaacaactctggc
gcatcgggcttcccatacaatcgatagattgtcgcacctgattgcccgacattatcgcgagcccatttatacccatataaatcagcatccat
gttggaatttaatcgcggcttcgagcaagacgtttcccgttgaatatggctcataacacccccttgtattactgtttatgtaagcagacagtttta
ttgttcatgatgatatatttttatcttgtgcaatgtaacatcagagattttgagacacaacgtg

EmptyT7	Empty vector that transcribes a 51-nt sequence from $P_{T7,strong}$

$P_{T7,strong}$ Terminator Kanamycin resistance cassette ColE1 origin

agatcaaaggatcttcttgagatcctttttttctgcgcgtaatctgctgcttgcaaacaaaaaaaccaccgctaccagcggtggtttgtttgcc
ggatcaagagctaccaactctttttccgaaggtaactggcttcagcagagcgcagataccaaatactgttcttctagtgtagccgtagttag
gccaccacttcaagaactctgtagcaccgcctacatacctcgctctgctaatcctgttaccagtggctgctgccagtggcgataagtcgtg
tcttaccgggttggactcaagacgatagttaccggataaggcgcagcggtcgggctgaacggggggttcgtgcacacagcccagcttg
gagcgaacgacctacaccgaactgagatacctacagcgtgagctatgagaaagcgccacgcttcccgaagggagaaaggcggaca
ggtatccggtaagcggcagggtcggaacaggagagcgcacgagggagcttccagggggaaacgcctggtatctttatagtcctgtcg
ggtttcgccacctctgacttgagcgtcgatttttgtgatgctcgtcaggggggcggagcctatggaaaaacgccagcaacgcgatcccg
cgaaattaatacgactcactatagggagagtcgaccggctgctaacaaagcccgaaaggaagctgagttggctgctgccaccgctgag
caataactagcataaccccttggggcctctaaacgggtcttgaggggttttttgctgaaagccaattctgattagaaaaactcatcgagcat
caaatgaaactgcaatttattcatatcaggattatcaataccatatttttgaaaaagccgtttctgtaatgaaggagaaaactcaccgaggca
gttccataggatggcaagatcctggtatcggtctgcgattccgactcgtccaacatcaatacaacctattaatttcccctcgtcaaaaataag
gttatcaagtgagaaatcaccatgagtgacgactgaatccggtgagaatggcaaaagcttatgcatttctttccagacttgttcaacaggcc
agccattacgctcgtcatcaaaatcactcgcatcaaccaaaccgttattcattcgtgattgcgcctgagcgagacgaaatacgcgatcgct
gttaaaaggacaattacaaacaggaatcgaatgcaaccggcgcaggaacactgccagcgcatcaacaatattttcacctgaatcaggat
attcttctaatacctggaatgctgttttcccggggatcgcagtggtgagtaaccatgcatcatcaggagtacggataaaatgcttgatggtc
ggaagaggcataaattccgtcagccagtttagtctgaccatctcatctgtaacatcattggcaacgctacctttgccatgtttcagaaacaac
tctggcgcatcgggcttcccatacaatcgatagattgtcgcacctgattgcccgacattatcgcgagcccatttatacccatataaatcagc
atccatgttggaatttaatcgcggcttcgagcaagacgtttcccgttgaatatggctcataacaccccttgtattactgtttatgtaagcagac
agttttattgttcatgatgatatatttttatcttgtgcaatgtaacatcagagattttgagacacaacgtg

EmptySigma70	Empty vector that transcribes a 51-nt sequence from $P_{\sigma70,strong}$

$P_{\sigma70,strong}$ Terminator Kanamycin resistance cassette ColE1 origin

agatcaaaggatcttcttgagatcctttttttctgcgcgtaatctgctgcttgcaaacaaaaaaaccaccgctaccagcggtggtttgtttgcc
ggatcaagagctaccaactctttttccgaaggtaactggcttcagcagagcgcagataccaaatactgttcttctagtgtagccgtagttag
gccaccacttcaagaactctgtagcaccgcctacatacctcgctctgctaatcctgttaccagtggctgctgccagtggcgataagtcgtg
tcttaccgggttggactcaagacgatagttaccggataaggcgcagcggtcgggctgaacggggggttcgtgcacacagcccagcttg
gagcgaacgacctacaccgaactgagatacctacagcgtgagctatgagaaagcgccacgcttcccgaagggagaaaggcggaca
ggtatccggtaagcggcagggtcggaacaggagagcgcacgagggagcttccagggggaaacgcctggtatctttatagtcctgtcg
ggtttcgccacctctgacttgagcgtcgatttttgtgatgctcgtcaggggggcggagcctatggaaaaacgccagcaacgcgatcccg
cgaaatttgacagctagctcagtcctaggtataatactagtgtcgaccggctgctaacaaagcccgaaaggaagctgagttggctgctgc
cagaagcttgggcccgaacaaaaactcatctcagaagaggatctgaatagcgccgtcgaccatcatcatcatcatcattgagtttaaacg
gtctccagcttggctgttttggcggatgagagaagattttcagcctgatacagattaaatcagaacgcagaagcggtctgataaaacagaa
tttgcctggcggcagtagcgcggtggtcccacctgaccccatgccgaactcagaagtgaaacgccgtagcgccgatggtagtgtggg
gtctccccatgcgagagtagggaactgccaggcatcaaataaaacgaaaggctcagtcgaaagactgggcctttcgttttatctgttgttt
gtcggtgaactctgaaagccaattctgattagaaaaactcatcgagcatcaaatgaaactgcaatttattcatatcaggattatcaataccat
atttttgaaaaagccgtttctgtaatgaaggagaaaactcaccgaggcagttccataggatggcaagatcctggtatcggtctgcgattcc
gactcgtccaacatcaatacaacctattaatttcccctcgtcaaaaataaggttatcaagtgagaaatcaccatgagtgacgactgaatccg
gtgagaatggcaaaagcttatgcatttctttccagacttgttcaacaggccagccattacgctcgtcatcaaaatcactcgcatcaaccaaa
ccgttattcattcgtgattgcgcctgagcgagacgaaatacgcgatcgctgttaaaaggacaattacaaacaggaatcgaatgcaaccgg
cgcaggaacactgccagcgcatcaacaatattttcacctgaatcaggatattcttctaatacctggaatgctgttttcccggggatcgcagt
ggtgagtaaccatgcatcatcaggagtacggataaaatgcttgatggtcggaagaggcataaattccgtcagccagtttagtctgaccatc
tcatctgtaacatcattggcaacgctacctttgccatgtttcagaaacaactctggcgcatcgggcttcccatacaatcgatagattgtcgca
cctgattgcccgacattatcgcgagcccatttatacccatataaatcagcatccatgttggaatttaatcgcggcttcgagcaagacgtttcc

cgttgaatatggctcataacacccttgtattactgtttatgtaagcagacagttttattgttcatgatgatatatttttatcttgtgcaatgtaacat<br>cagagattttgagacacaacgtg	

$P_{T7,strong}$ 3WJdB	Plasmid encoding 3WJDB expression under $P_{T7,strong}$
$P_{T7,strong}$ 3WJdB Terminator Kanamycin resistance cassette ColE1 origin<br><br>Agatcaaaggatcttcttgagatcctttttttctgcgcgtaatctgctgcttgcaaacaaaaaaaccaccgctaccagcggtggtttgtttgc<br>cggatcaagagctaccaactctttttccgaaggtaactggcttcagcagagcgcagataccaaatactgttcttctagtgtagccgtagtta<br>ggccaccacttcaagaactctgtagcaccgcctacatacctcgctctgctaatcctgttaccagtggctgctgccagtggcgataagtcgt<br>gtcttaccgggttggactcaagacgatagttaccggataaggcgcagcggtcgggctgaacggggggttcgtgcacacagcccagctt<br>ggagcgaacgacctacaccgaactgagatacctacagcgtgagctatgagaaagcgccacgcttcccgaagggagaaaggcggac<br>aggtatccggtaagcggcagggtcggaacaggagagcgcacgagggagcttccagggggaaacgcctggtatctttatagtcctgtc<br>gggtttcgccacctctgacttgagcgtcgatttttgtgatgctcgtcaggggggcggagcctatggaaaaacgccagcaacgcgatccc<br>gcgaaattaatacgactcactatagggagacccacatactctgatgatccgagacggtcgggtccagatattcgtatctgtcgagtagagt<br>gtgggctcggatcattcatggcaagagacggtcgggtccagatattcgtatctgtcgagtagagtgtgggctcttgccatgtgtatgtggg<br>tagcataaccccttggggcctctaaacgggtcttgaggggttttttgctgaaagccaattctgattagaaaaactcatcgagcatcaaatga<br>aactgcaatttattcatatcaggattatcaataccatatttttgaaaaagccgtttctgtaatgaaggagaaaactcaccgaggcagttccata<br>ggatggcaagatcctggtatcggtctgcgattccgactcgtccaacatcaatacaacctattaatttcccctcgtcaaaaataaggttatcaa<br>gtgagaaatcaccatgagtgacgactgaatccggtgagaatggcaaaagcttatgcatttctttccagacttgttcaacaggccagccatt<br>acgctcgtcatcaaaatcactcgcatcaaccaaaccgttattcattcgtgattgcgcctgagcgagacgaaatacgcgatcgctgttaaaa<br>ggacaattacaaacaggaatcgaatgcaaccggcgcaggaacactgccagcgcatcaacaatattttcacctgaatcaggatattcttct<br>aatacctggaatgctgttttcccggggatcgcagtggtgagtaaccatgcatcatcaggagtacggataaaatgcttgatggtcggaaga<br>ggcataaattccgtcagccagtttagtctgaccatctcatctgtaacatcattggcaacgctacctttgccatgtttcagaaacaactctggc<br>gcatcgggcttcccatacaatcgatagattgtcgcacctgattgcccgacattatcgcgagcccatttatacccatataaatcagcatccat<br>gttggaatttaatcgcggcttcgagcaagacgtttcccgttgaatatggctcataacacccttgtattactgtttatgtaagcagacagtttta<br>ttgttcatgatgatatatttttatcttgtgcaatgtaacatcagagattttgagacacaacgtg	

$P_{T7,weak}$ 3WJdB	Plasmid encoding 3WJDB expression under $P_{T7,weak}$
$P_{T7,weak}$ 3WJdB Terminator Kanamycin resistance cassette ColE1 origin<br><br>agatcaaaggatcttcttgagatcctttttttctgcgcgtaatctgctgcttgcaaacaaaaaaaccaccgctaccagcggtggtttgtttgcc<br>ggatcaagagctaccaactctttttccgaaggtaactggcttcagcagagcgcagataccaaatactgttcttctagtgtagccgtagttag<br>gccaccacttcaagaactctgtagcaccgcctacatacctcgctctgctaatcctgttaccagtggctgctgccagtggcgataagtcgtg<br>tcttaccgggttggactcaagacgatagttaccggataaggcgcagcggtcgggctgaacggggggttcgtgcacacagcccagcttg<br>gagcgaacgacctacaccgaactgagatacctacagcgtgagctatgagaaagcgccacgcttcccgaagggagaaaggcggaca<br>ggtatccggtaagcggcagggtcggaacaggagagcgcacgagggagcttccagggggaaacgcctggtatctttatagtcctgtcg<br>ggtttcgccacctctgacttgagcgtcgatttttgtgatgctcgtcaggggggcggagcctatggaaaaacgccagcaacgcgatcccg<br>cgaaatTAATACGACTCACTAAAGGgagacccacatactctgatgatccgagacggtcgggtccagatattcgtatct<br>gtcgagtagagtgtgggctcggatcattcatggcaagagacggtcgggtccagatattcgtatctgtcgagtagagtgtgggctcttgcc<br>atgtgtatgtgggagcataaccccttggggcctctaaacgggtcttgaggggttttttgctgaaagccaattctgattagaaaaactcatcg<br>agcatcaaatgaaactgcaatttattcatatcaggattatcaataccatatttttgaaaaagccgtttctgtaatgaaggagaaaactcaccg<br>aggcagttccataggatggcaagatcctggtatcggtctgcgattccgactcgtccaacatcaatacaacctattaatttcccctcgtcaaa<br>aataaggttatcaagtgagaaatcaccatgagtgacgactgaatccggtgagaatggcaaaagcttatgcatttctttccagacttgttcaa<br>caggccagccattacgctcgtcatcaaaatcactcgcatcaaccaaaccgttattcattcgtgattgcgcctgagcgagacgaaatacgc<br>gatcgctgttaaaaggacaattacaaacaggaatcgaatgcaaccggcgcaggaacactgccagcgcatcaacaatattttcacctgaa	

tcaggatattcttctaatacctggaatgctgttttcccggggatcgcagtggtgagtaaccatgcatcatcaggagtacggataaaatgctt
gatggtcggaagaggcataaattccgtcagccagtttagtctgaccatctcatctgtaacatcattggcaacgctacctttgccatgtttcag
aaacaactctggcgcatcgggcttcccatacaatcgatagattgtcgcacctgattgcccgacattatcgcgagcccatttatacccatata
aatcagcatccatgttggaatttaatcgcggcttcgagcaagacgtttcccgttgaatatggctcataacacccccttgtattactgtttatgta
agcagacagttttattgttcatgatgatatatttttatcttgtgcaatgtaacatcagagattttgagacacaacgtg

$P_{\sigma70,strong}$ 3WJdB	Plasmid encoding 3WJDB expression under $P_{\sigma70,strong}$

$P_{\sigma70,strong}$ 3WJdB Terminator Kanamycin resistance cassette ColE1 origin

agatcaaaggatcttcttgagatcctttttttctgcgcgtaatctgctgcttgcaaacaaaaaaaccaccgctaccagcggtggtttgtttgcc
ggatcaagagctaccaactctttttccgaaggtaactggcttcagcagagcgcagataccaaatactgttcttctagtgtagccgtagttag
gccaccacttcaagaactctgtagcaccgcctacatacctcgctctgctaatcctgttaccagtggctgctgccagtggcgataagtcgtg
tcttaccgggttggactcaagacgatagttaccggataaggcgcagcggtcgggctgaacggggggttcgtgcacacagcccagcttg
gagcgaacgacctacaccgaactgagatacctacagcgtgagctatgagaaagcgccacgcttcccgaagggagaaaggcggaca
ggtatccggtaagcggcagggtcggaacaggagagcgcacgagggagcttccagggggaaacgcctggtatctttatagtcctgtcg
ggtttcgccacctctgacttgagcgtcgatttttgtgatgctcgtcaggggggcggagcctatggaaaaacgccagcaacgcgatcccg
cgaaatttgacagctagctcagtcctaggtataatactagtcccacatactctgatgatccgagacggtcgggtccagatattcgtatctgt
cgagtagagtgtgggctcggatcattcatggcaagagacggtcgggtccagatattcgtatctgtcgagtagagtgtgggctcttgccat
gtgtatgtggggaagcttgggcccgaacaaaaactcatctcagaagaggatctgaatagcgccgtcgaccatcatcatcatcatcattga
gtttaaacggtctccagcttggctgttttggcggatgagagaagattttcagcctgatacagattaaatcagaacgcagaagcggtctgat
aaaacagaatttgcctggcggcagtagcgcggtggtcccacctgaccccatgccgaactcagaagtgaaacgccgtagcgccgatgg
tagtgtggggtctccccatgcgagagtagggaactgccaggcatcaaataaaacgaaaggctcagtcgaaagactgggcctttcgtttt
atctgttgtttgtcggtgaactctgaaagccaattctgattagaaaaactcatcgagcatcaaatgaaactgcaatttattcatatcaggattat
caataccatatttttgaaaaagccgtttctgtaatgaaggagaaaactcaccgaggcagttccataggatggcaagatcctggtatcggtc
tgcgattccgactcgtccaacatcaatacaacctattaatttcccctcgtcaaaaataaggttatcaagtgagaaatcaccatgagtgacga
ctgaatccggtgagaatggcaaaagcttatgcatttctttccagacttgttcaacaggccagccattacgctcgtcatcaaaatcactcgca
tcaaccaaaccgttattcattcgtgattgcgcctgagcgagacgaaatacgcgatcgctgttaaaaggacaattacaaacaggaatcgaa
tgcaaccggcgcaggaacactgccagcgcatcaacaatattttcacctgaatcaggatattcttctaatacctggaatgctgttttcccggg
gatcgcagtggtgagtaaccatgcatcatcaggagtacggataaaatgcttgatggtcggaagaggcataaattccgtcagccagtttag
tctgaccatctcatctgtaacatcattggcaacgctacctttgccatgtttcagaaacaactctggcgcatcgggcttcccatacaatcgata
gattgtcgcacctgattgcccgacattatcgcgagcccatttatacccatataaatcagcatccatgttggaatttaatcgcggcttcgagca
agacgtttcccgttgaatatggctcataacacccccttgtattactgtttatgtaagcagacagttttattgttcatgatgatatatttttatcttgtg
caatgtaacatcagagattttgagacacaacgtg

$P_{\sigma70,weak}$ 3WJdB	Plasmid encoding 3WJDB expression under $P_{\sigma70,weak}$

$P_{\sigma70,weak}$ 3WJdB Terminator Kanamycin resistance cassette ColE1 origin

agatcaaaggatcttcttgagatcctttttttctgcgcgtaatctgctgcttgcaaacaaaaaaaccaccgctaccagcggtggtttgtttgcc
ggatcaagagctaccaactctttttccgaaggtaactggcttcagcagagcgcagataccaaatactgttcttctagtgtagccgtagttag
gccaccacttcaagaactctgtagcaccgcctacatacctcgctctgctaatcctgttaccagtggctgctgccagtggcgataagtcgtg
tcttaccgggttggactcaagacgatagttaccggataaggcgcagcggtcgggctgaacggggggttcgtgcacacagcccagcttg
gagcgaacgacctacaccgaactgagatacctacagcgtgagctatgagaaagcgccacgcttcccgaagggagaaaggcggaca
ggtatccggtaagcggcagggtcggaacaggagagcgcacgagggagcttccagggggaaacgcctggtatctttatagtcctgtcg
ggtttcgccacctctgacttgagcgtcgatttttgtgatgctcgtcaggggggcggagcctatggaaaaacgccagcaacgcgatcccg
cgaaattttacggctagctcagtcctaggtactatgctagccccacatactctgatgatccgagacggtcgggtccagatattcgtatctgt
cgagtagagtgtgggctcggatcattcatggcaagagacggtcgggtccagatattcgtatctgtcgagtagagtgtgggctcttgccat
gtgtatgtggggaagcttgggcccgaacaaaaactcatctcagaagaggatctgaatagcgccgtcgaccatcatcatcatcatcattga

gtttaaacggtctccagcttggctgttttggcggatgagagaagattttcagcctgatacagattaaatcagaacgcagaagcggtctgat
aaaacagaatttgcctggcggcagtagcgcggtggtcccacctgaccccatgccgaactcagaagtgaaacgccgtagcgccgatgg
tagtgtggggtctccccatgcgagagtagggaactgccaggcatcaaataaaacgaaaggctcagtcgaaagactgggcctttcgtttt
atctgttgtttgtcggtgaactctgaaagccaattctgattagaaaaactcatcgagcatcaaatgaaactgcaatttattcatatcaggattat
caataccatatttttgaaaaagccgtttctgtaatgaaggagaaaactcaccgaggcagttccataggatggcaagatcctggtatcggtc
tgcgattccgactcgtccaacatcaatacaacctattaatttcccctcgtcaaaaataaggttatcaagtgagaaatcaccatgagtgacga
ctgaatccggtgagaatggcaaaagcttatgcatttctttccagacttgttcaacaggccagccattacgctcgtcatcaaaatcactcgca
tcaaccaaaccgttattcattcgtgattgcgcctgagcgagacgaaatacgcgatcgctgttaaaaggacaattacaaacaggaatcgaa
tgcaaccggcgcaggaacactgccagcgcatcaacaatattttcacctgaatcaggatattcttctaatacctggaatgctgttttcccggg
gatcgcagtggtgagtaaccatgcatcatcaggagtacggataaaatgcttgatggtcggaagaggcataaattccgtcagccagtttag
tctgaccatctcatctgtaacatcattggcaacgctacctttgccatgtttcagaaacaactctggcgcatcgggcttcccatacaatcgata
gattgtcgcacctgattgcccgacattatcgcgagcccatttatacccatataaatcagcatccatgttggaatttaatcgcggcttcgagca
agacgtttcccgttgaatatggctcataacacccccttgtattactgtttatgtaagcagacagttttattgttcatgatgatatatttttatcttgtg
caatgtaacatcagagattttgagacacaacgtg

$P_{T7,strong}$ mRFP	Plasmid encoding mRFP expression under $P_{T7,strong}$.

$P_{T7,strong}$ Stability hairpin RBS mRFP Terminator Kanamycin resistance cassette ColE1 origin

agatcaaaggatcttcttgagatcctttttttctgcgcgtaatctgctgcttgcaaacaaaaaaaccaccgctaccagcggtggtttgtttgcc
ggatcaagagctaccaactctttttccgaaggtaactggcttcagcagagcgcagataccaaatactgttcttctagtgtagccgtagttag
gccaccacttcaagaactctgtagcaccgcctacatacctcgctctgctaatcctgttaccagtggctgctgccagtggcgataagtcgtg
tcttaccgggttggactcaagacgatagttaccggataaggcgcagcggtcgggctgaacggggggttcgtgcacacagcccagcttg
gagcgaacgacctacaccgaactgagatacctacagcgtgagctatgagaaagcgccacgcttcccgaagggagaaaggcggaca
ggtatccggtaagcggcagggtcggaacaggagagcgcacgagggagcttccagggggaaacgcctggtatctttatagtcctgtcg
ggtttcgccacctctgacttgagcgtcgatttttgtgatgctcgtcaggggggcggagcctatggaaaaacgccagcaacgcgatcccg
cgaaattaatacgactcactatagggagaccacaacggtttccctctagaaataattttgtttaactttaagaaggagatatacatatggcttc
ctccgaagacgttatcaaagagttcatgcgtttcaaagttcgtatggaaggttccgttaacggtcacgagttcgaaatcgaaggtgaaggt
gaaggtcgtccgtacgaaggtacccagaccgctaaactgaaagttaccaaaggtggtccgctgccgttcgcttgggacatcctgtcccc
gcagttccagtacggttccaaagcttacgttaaacacccggctgacatcccggactacctgaaactgtccttcccggaaggtttcaaatgg
gaacgtgttatgaacttcgaagacggtggtgttgttaccgttacccaggactcctccctgcaagacggtgagttcatctacaaagttaaact
gcgtggtaccaacttcccgtccgacggtccggttatgcagaaaaaaaccatgggttgggaagcttccaccgaacgtatgtacccggaag
acggtgctctgaaaggtgaaatcaaaatgcgtctgaaactgaaagacggtggtcactacgacgctgaagttaaaaccacctacatggct
aaaaaaccggttcagctgccgggtgcttacaaaaccgacatcaaactggacatcacctcccacaacgaagactacaccatcgttgaaca
gtacgaacgtgctgaaggtcgtcactccaccggtgcttaatagtcgaccggctgctaacaaagcccgaaaggaagctgagttggctgct
gccaccgctgagcaataactagcataaccccttggggcctctaaacgggtcttgaggggttttttgctgaaagccaattctgattagaaaa
actcatcgagcatcaaatgaaactgcaatttattcatatcaggattatcaataccatatttttgaaaaagccgtttctgtaatgaaggagaaaa
ctcaccgaggcagttccataggatggcaagatcctggtatcggtctgcgattccgactcgtccaacatcaatacaacctattaatttcccct
cgtcaaaaataaggttatcaagtgagaaatcaccatgagtgacgactgaatccggtgagaatggcaaaagcttatgcatttctttccagac
ttgttcaacaggccagccattacgctcgtcatcaaaatcactcgcatcaaccaaaccgttattcattcgtgattgcgcctgagcgagacga
aatacgcgatcgctgttaaaaggacaattacaaacaggaatcgaatgcaaccggcgcaggaacactgccagcgcatcaacaatattttc
acctgaatcaggatattcttctaatacctggaatgctgttttcccggggatcgcagtggtgagtaaccatgcatcatcaggagtacggataa
aatgcttgatggtcggaagaggcataaattccgtcagccagtttagtctgaccatctcatctgtaacatcattggcaacgctacctttgccat
gtttcagaaacaactctggcgcatcgggcttcccatacaatcgatagattgtcgcacctgattgcccgacattatcgcgagcccatttatac
ccatataaatcagcatccatgttggaatttaatcgcggcttcgagcaagacgtttcccgttgaatatggctcataacacccccttgtattactgt
ttatgtaagcagacagttttattgttcatgatgatatatttttatcttgtgcaatgtaacatcagagattttgagacacaacgtg

$P_{T7,strong}$ LacI-sfGFP	Plasmid encoding LacI-sfGFP expression under $P_{T7,strong}$.

$P_{T7,strong}$ Stability hairpin RBS his6-tag LacI Linker sfGFP Terminator Kanamycin resistance cassette ColE1 origin

agatcaaaggatcttcttgagatcctttttttctgcgcgtaatctgctgcttgcaaacaaaaaaaccaccgctaccagcggtggtttgtttgcc
ggatcaagagctaccaactctttttccgaaggtaactggcttcagcagagcgcagataccaaatactgttcttctagtgtagccgtagttag
gccaccacttcaagaactctgtagcaccgcctacatacctcgctctgctaatcctgttaccagtggctgctgccagtggcgataagtcgtg
tcttaccgggttggactcaagacgatagttaccggataaggcgcagcggtcgggctgaacggggggttcgtgcacacagcccagcttg
gagcgaacgacctacaccgaactgagatacctacagcgtgagctatgagaaagcgccacgcttcccgaagggagaaaggcggaca
ggtatccggtaagcggcagggtcggaacaggagagcgcacgagggagcttccagggggaaacgcctggtatctttatagtcctgtcg
ggtttcgccacctctgacttgagcgtcgatttttgtgatgctcgtcaggggggcggagcctatggaaaaacgccagcaacgcgatcccg
cgaaattaatacgactcactatagggagaccacaacggtttccctctagaaataattttgtttaactttaagaaggagatatacatATGcat
catcaccaccaccatAAACCAGTAACGTTATACGATGTCGCAGAGTATGCCGGTGTCTCTT
ATCAGACCGTTTCCCGCGTGGTGAACCAGGCCAGCCACGTTTCTGCGAAAACGCG
GGAAAAAGTGGAAGCGGCGATGGCGGAGCTGAATTACATTCCCAACCGCGTGGC
ACAACAACTGGCGGGCAAACAGTCGTTGCTGATTGGCGTTGCCACCTCCAGTCTG
GCCCTGCACGCGCCGTCGCAAATTGTCGCGGCGATTAAATCTCGCGCCGATCAAC
TGGGTGCCAGCGTGGTGGTGTCGATGGTAGAACGAAGCGGCGTCGAAGCCTGTAA
AGCGGCGGTGCACAATCTTCTCGCGCAACGCGTCAGTGGGCTGATCATTAACTAT
CCGCTGGATGACCAGGATGCCATTGCTGTGGAAGCTGCCTGCACTAATGTTCCGG
CGTTATTTCTTGATGTCTCTGACCAGACACCCATCAACAGTATTATTTTCTCCCATG
AAGACGGTACGCGACTGGGCGTGGAGCATCTGGTCGCATTGGGTCACCAGCAAAT
CGCGCTGTTAGCGGGCCCATTAAGTTCTGTCTCGGCGCGTCTGCGTCTGGCTGGCT
GGCATAAATATCTCACTCGCAATCAAATTCAGCCGATAGCGGAACGGGAAGGCGA
CTGGAGTGCCATGTCCGGTTTTCAACAAACCATGCAAATGCTGAATGAGGGCATC
GTTCCCACTGCGATGCTGGTTGCCAACGATCAGATGGCGCTGGGCGCAATGCGCG
CCATTACCGAGTCCGGGCTGCGCGTTGGTGCGGATATCTCGGTAGTGGGATACGA
CGATACCGAAGACAGCTCATGTTATATCCCGCCGTTAACCACCATCAAACAGGAT
TTTCGCCTGCTGGGGCAAACCAGCGTGGACCGCTTGCTGCAACTCTCTCAGGGCCA
GGCGGTGAAGGGCAATCAGCTGTTGCCCGTCTCACTGGTGAAAAGAAAAACCACC
CTGGCGCCCAATACGCAAACCGCCTCTCCCCGCGCGTTGGCCGATTCATTAATGCA
GCTGGCACGACAGGTTTCCCGAggtggcagtgggagtagcGGTGGTTCAGGCAGCGGGTCA
AGCGGCGGATCTGGAAGCTCAggaggtagcgggagtggcagcagtAGCAAAGGTGAAGAACT
GTTTACCGGCGTTGTGCCGATTCTGGTGGAACTGGATGGCGATGTGAACGGTCAC
AAATTCAGCGTGCGTGGTGAAGGTGAAGGCGATGCCACGATTGGCAAACTGACGC
TGAAATTTATCTGCACCACCGGCAAACTGCCGGTGCCGTGGCCGACGCTGGTGAC
CACCCTGACCTATGGCGTTCAGTGTTTTAGTCGCTATCCGGATCACATGAAACGTC
ACGATTTCTTTAAATCTGCAATGCCGGAAGGCTATGTGCAGGAACGTACGATTAG
CTTTAAAGATGATGGCAAATATAAAACGCGCGCCGTTGTGAAATTTGAAGGCGAT
ACCCTGGTGAACCGCATTGAACTGAAAGGCACGGATTTTAAAGAAGATGGCAATA
TCCTGGGCCATAAACTGGAATACAACTTTAATAGCCATAATGTTTATATTACGGCG
GATAAACAGAAAAATGGCATCAAAGCGAATTTTACCGTTCGCCATAACGTTGAAG
ATGGCAGTGTGCAGCTGGCAGATCATTATCAGCAGAATACCCCGATTGGTGATGG
TCCGGTGCTGCTGCCGGATAATCATTATCTGAGCACGCAGACCGTTCTGTCTAAAG

ATCCGAACGAAAAAGGCACGCGGACCACATGGTTCTGCACGAATATGTGAATGC
GGCAGGTATTACGTGGAGCCATCCGCAGTTCGAAAAATAAgtcgaccggctgctaacaaagccc
gaaaggaagctgagttggctgctgccaccgctgagcaataactagcataaccccttggggcctctaaacgggtcttgaggggttttttgct
gaaagccaattctgattagaaaaactcatcgagcatcaaatgaaactgcaatttattcatatcaggattatcaataccatatttttgaaaaagc
cgtttctgtaatgaaggagaaaactcaccgaggcagttccataggatggcaagatcctggtatcggtctgcgattccgactcgtccaacat
caatacaacctattaatttcccctcgtcaaaaataaggttatcaagtgagaaatcaccatgagtgacgactgaatccggtgagaatggcaa
aagcttatgcatttctttccagacttgttcaacaggccagccattacgctcgtcatcaaaatcactcgcatcaaccaaaccgttattcattcgt
gattgcgcctgagcgagacgaaatacgcgatcgctgttaaaaggacaattacaaacaggaatcgaatgcaaccggcgcaggaacact
gccagcgcatcaacaatattttcacctgaatcaggatattcttctaatacctggaatgctgttttcccggggatcgcagtggtgagtaaccat
gcatcatcaggagtacggataaaatgcttgatggtcggaagaggcataaattccgtcagccagtttagtctgaccatctcatctgtaacatc
attggcaacgctacctttgccatgtttcagaaacaactctggcgcatcgggcttcccatacaatcgatagattgtcgcacctgattgcccga
cattatcgcgagcccatttatacccatataaatcagcatccatgttggaatttaatcgcggcttcgagcaagacgtttcccgttgaatatggct
cataacaccccttgtattactgtttatgtaagcagacagttttattgttcatgatgatatatttttatcttgtgcaatgtaacatcagagattttgag
acacaacgtg

$P_{\sigma 70,weak}$ LacO mRFP	Plasmid encoding mRFP expression under $P_{\sigma 70,weak}$ and LacO operator

$P_{\sigma 70,weak}$ LacO Stability hairpin RBS mRFP Terminator Kanamycin resistance cassette ColE1 origin

agatcaaaggatcttcttgagatcctttttttctgcgcgtaatctgctgcttgcaaacaaaaaaaccaccgctaccagcggtggtttgtttgcc
ggatcaagagctaccaactctttttccgaaggtaactggcttcagcagagcgcagataccaaatactgttcttctagtgtagccgtagttag
gccaccacttcaagaactctgtagcaccgcctacatacctcgctctgctaatcctgttaccagtggctgctgccagtggcgataagtcgtg
tcttaccgggttggactcaagacgatagttaccggataaggcgcagcggtcgggctgaacggggggttcgtgcacacagcccagcttg
gagcgaacgacctacaccgaactgagatacctacagcgtgagctatgagaaagcgccacgcttcccgaagggagaaaggcggaca
ggtatccggtaagcggcagggtcggaacaggagagcgcacgagggagcttccagggggaaacgcctggtatctttatagtcctgtcg
ggtttcgccacctctgacttgagcgtcgatttttgtgatgctcgtcaggggggcggagcctatggaaaaacgccagcaacgcgatcccg
cgaaatttacggctagctcagtcctaggtactatgctagcgagaggaattgtgagcggataacaattccacaacggtttccctctagaaat
aattttgtttaactttaagaaggagatatacatatggcttcctccgaagacgttatcaaagagttcatgcgtttcaaagttcgtatggaaggttc
cgttaacggtcacgagttcgaaatcgaaggtgaaggtgaaggtcgtccgtacgaaggtacccagaccgctaaactgaaagttaccaaa
ggtggtccgctgccgttcgcttgggacatcctgtccccgcagttccagtacggttccaaagcttacgttaaacacccggctgacatcccg
gactacctgaaactgtccttcccggaaggtttcaaatgggaacgtgttatgaacttcgaagacggtggtgttgttaccgttacccaggactc
ctccctgcaagacggtgagttcatctacaaagttaaactgcgtggtaccaacttcccgtccgacggtccggttatgcagaaaaaaaccat
gggttgggaagcttccaccgaacgtatgtacccggaagacggtgctctgaaaggtgaaatcaaaatgcgtctgaaactgaaagacggt
ggtcactacgacgctgaagttaaaaccacctacatggctaaaaaaccggttcagctgccgggtgcttacaaaaccgacatcaaactgga
catcacctcccacaacgaagactacaccatcgttgaacagtacgaacgtgctgaaggtcgtcactccaccggtgcttaatagtcgaccg
gctgctaacaaagcccgaaaggaagctgagttggctgctgccaccgctgagcaataactagcataaccccttggggcctctaaacggg
tcttgaggggttttttgctgaaagccaattctgattagaaaaactcatcgagcatcaaatgaaactgcaatttattcatatcaggattatcaata
ccatatttttgaaaaagccgtttctgtaatgaaggagaaaactcaccgaggcagttccataggatggcaagatcctggtatcggtctgcga
ttccgactcgtccaacatcaatacaacctattaatttcccctcgtcaaaaataaggttatcaagtgagaaatcaccatgagtgacgactgaat
ccggtgagaatggcaaaagcttatgcatttctttccagacttgttcaacaggccagccattacgctcgtcatcaaaatcactcgcatcaacc
aaaccgttattcattcgtgattgcgcctgagcgagacgaaatacgcgatcgctgttaaaaggacaattacaaacaggaatcgaatgcaac
cggcgcaggaacactgccagcgcatcaacaatattttcacctgaatcaggatattcttctaatacctggaatgctgttttcccggggatcgc
agtggtgagtaaccatgcatcatcaggagtacggataaaatgcttgatggtcggaagaggcataaattccgtcagccagtttagtctgac
catctcatctgtaacatcattggcaacgctacctttgccatgtttcagaaacaactctggcgcatcgggcttcccatacaatcgatagattgt
cgcacctgattgcccgacattatcgcgagcccatttatacccatataaatcagcatccatgttggaatttaatcgcggcttcgagcaagacg

tttcccgttgaatatggctcataacaccccttgtattactgtttatgtaagcagacagttttattgttcatgatgatatatttttatcttgtgcaatgt<br>aacatcagagattttgagacacaacgtg

$P_{T7,strong}$ switchB LacZ	Plasmid encoding LacZ expression under $P_{T7,strong}$ and switchB
$P_{T7,strong}$ switchB LacZ Terminator Kanamycin resistance cassette ColE1 origin<br><br>agatcaaaggatcttcttgagatcctttttttctgcgcgtaatctgctgcttgcaaacaaaaaaaccaccgctaccagcggtggtttgtttgcc<br>ggatcaagagctaccaactctttttccgaaggtaactggcttcagcagagcgcagataccaaatactgttcttctagtgtagccgtagttag<br>gccaccacttcaagaactctgtagcaccgcctacatacctcgctctgctaatcctgttaccagtggctgctgccagtggcgataagtcgtg<br>tcttaccgggttggactcaagacgatagttaccggataaggcgcagcggtcgggctgaacggggggttcgtgcacacagcccagcttg<br>gagcgaacgacctacaccgaactgagatacctacagcgtgagctatgagaaagcgccacgcttcccgaagggagaaaggcggaca<br>ggtatccggtaagcggcagggtcggaacaggagagcgcacgagggagcttccagggggaaacgcctggtatctttatagtcctgtcg<br>ggtttcgccacctctgacttgagcgtcgatttttgtgatgctcgtcaggggggcggagcctatggaaaaacgccagcaacgcgatcccg<br>cgaaattaatacgactcactatagggagaGGGTATAAGTAAATCGCTTGCTGTATGTCGTTAAACAG<br>AGGAGATAACGAATGACAGCAAGCAACCTGGCGGCAGCGCAAAAG<br>atgACCATGATTACGGATTCACTGGCCGTCGTTTTACAACGTCGTGACTGGGAAAAC<br>CCTGGCGTTACCCAACTTAATCGCCTTGCAGCACATCCCCCTTTCGCCAGCTGGCG<br>TAATAGCGAAGAGGCCCGCACCGATCGCCCTTCCCAACAGTTGCGCAGCCTGAAT<br>GGCGAATGGCGCTTTGCCTGGTTTCCGGCACCAGAAGCGGTGCCGGAAAGCTGGC<br>TGGAGTGCGATCTTCCTGAGGCCGATACTGTCGTCGTCCCCTCAAACTGGCAGATG<br>CACGGTTACGATGCGCCCATCTACACCAACGTGACCTATCCCATTACGGTCAATCC<br>GCCGTTTGTTCCCACGGAGAATCCGACGGGTTGTTACTCGCTCACATTTAATGTTG<br>ATGAAAGCTGGCTACAGGAAGGCCAGACGCGAATTATTTTTGATGGCGTTAACTC<br>GGCGTTTCATCTGTGGTGCAACGGGCGCTGGGTCGGTTACGGCCAGGACAGTCGT<br>TTGCCGTCTGAATTTGACCTGAGCGCATTTTTACGCGCCGGAGAAAACCGCCTCGC<br>GGTGATGGTGCTGCGCTGGAGTGACGGCAGTTATCTGGAAGATCAGGATATGTGG<br>CGGATGAGCGGCATTTTCCGTGACGTCTCGTTGCTGCATAAACCGACTACACAAAT<br>CAGCGATTTCCATGTTGCCACTCGCTTTAATGATGATTTCAGCCGCGCTGTACTGG<br>AGGCTGAAGTTCAGATGTGCGGCGAGTTGCGTGACTACCTACGGGTAACAGTTTC<br>TTTATGGCAGGGTGAAACGCAGGTCGCCAGCGGCACCGCGCCTTTCGGCGGTGAA<br>ATTATCGATGAGCGTGGTGGTTATGCCGATCGCGTCACACTACGTCTGAACGTCGA<br>AAACCCGAAACTGTGGAGCGCCGAAATCCCGAATCTCTATCGTGCGGTGGTTGAA<br>CTGCACACCGCCGACGGCACGCTGATTGAAGCAGAAGCCTGCGATGTCGGTTTCC<br>GCGAGGTGCGGATTGAAAATGGTCTGCTGCTGCTGAACGGCAAGCCGTTGCTGAT<br>TCGAGGCGTTAACCGTCACGAGCATCATCCTCTGCATGGTCAGGTCATGGATGAG<br>CAGACGATGGTGCAGGATATCCTGCTGATGAAGCAGAACAACTTTAACGCCGTGC<br>GCTGTTCGCATTATCCGAACCATCCGCTGTGGTACACGCTGTGCGACCGCTACGGC<br>CTGTATGTGGTGGATGAAGCCAATATTGAAACCCACGGCATGGTGCCAATGAATC<br>GTCTGACCGATGATCCGCGCTGGCTACCGGCGATGAGCGAACGCGTAACGCGAAT<br>GGTGCAGCGCGATCGTAATCACCCGAGTGTGATCATCTGGTCGCTGGGGAATGAA<br>TCAGGCCACGGCGCTAATCACGACGCGCTGTATCGCTGGATCAAATCTGTCGATC<br>CTTCCCGCCCGGTGCAGTATGAAGGCGGCGGAGCCGACACCACGGCCACCGATAT<br>TATTTGCCCGATGTACGCGCGCGTGGATGAAGACCAGCCCTTCCCGGCTGTGCCG<br>AAATGGTCCATCAAAAAATGGCTTTCGCTACCTGGAGAGACGCGCCCGCTGATCC<br>TTTGCGAATACGCCCACGCGATGGGTAACAGTCTTGGCGGTTTCGCTAAATACTGG<br>CAGGCGTTTCGTCAGTATCCCCGTTTACAGGGCGGCTTCGTCTGGGACTGGGTGGA	

TCAGTCGCTGATTAAATATGATGAAAACGGCAACCCGTGGTCGGCTTACGGCGGT<br>GATTTTGGCGATACGCCGAACGATCGCCAGTTCTGTATGAACGGTCTGGTCTTTGC<br>CGACCGCACGCCGCATCCAGCGCTGACGGAAGCAAAACACCAGCAGCAGTTTTTC<br>CAGTTCCGTTTATCCGGGCAAACCATCGAAGTGACCAGCGAATACCTGTTCCGTCA<br>TAGCGATAACGAGCTCCTGCACTGGATGGTGGCGCTGGATGGTAAGCCGCTGGCA<br>AGCGGTGAAGTGCCTCTGGATGTCGCTCCACAAGGTAAACAGTTGATTGAACTGC<br>CTGAACTACCGCAGCCGGAGAGCGCCGGGCAACTCTGGCTCACAGTACGCGTAGT<br>GCAACCGAACGCGACCGCATGGTCAGAAGCCGGACACATCAGCGCCTGGCAGCA<br>GTGGCGTCTGGCTGAAAACCTCAGCGTGACACTCCCCGCCGCGTCCCACGCCATC<br>CCGCATCTGACCACCAGCGAAATGGATTTTTGCATCGAGCTGGGTAATAAGCGTT<br>GGCAATTTAACCGCCAGTCAGGCTTTCTTTCACAGATGTGGATTGGCGATAAAAA<br>ACAACTGCTGACGCCGCTGCGCGATCAGTTCACCCGTGCACCGCTGGATAACGAC<br>ATTGGCGTAAGTGAAGCGACCCGCATTGACCCTAACGCCTGGGTCGAACGCTGGA<br>AGGCGGCGGGCCATTACCAGGCCGAAGCAGCGTTGTTGCAGTGCACGGCAGATAC<br>ACTTGCTGATGCGGTGCTGATTACGACCGCTCACGCGTGGCAGCATCAGGGGAAA<br>ACCTTATTTATCAGCCGGAAAACCTACCGGATTGATGGTAGTGGTCAAATGGCGA<br>TTACCGTTGATGTTGAAGTGGCGAGCGATACACCGCATCCGGCGCGGATTGGCCT<br>GAACTGCCAGCTGGCGCAGGTAGCAGAGCGGGTAAACTGGCTCGGATTAGGGCC<br>GCAAGAAAACTATCCCGACCGCCTTACTGCCGCCTGTTTTGACCGCTGGGATCTGC<br>CATTGTCAGACATGTATACCCCGTACGTCTTCCCGAGCGAAAACGGTCTGCGCTGC<br>GGGACGCGCGAATTGAATTATGGCCCACACCAGTGGCGCGGCGACTTCCAGTTCA<br>ACATCAGCCGCTACAGTCAACAGCAACTGATGGAAACCAGCCATCGCCATCTGCT<br>GCACGCGGAAGAAGGCACATGGCTGAATATCGACGGTTTCCATATGGGGATTGGT<br>GGCGACGACTCCTGGAGCCCGTCAGTATCGGCGGAATTCCAGCTGAGCGCCGGTC<br>GCTACCATTACCAGTTGGTCTGGTGTCAAAAAtaagtcgaccggctgctaacaaagcccgaaaggaagc<br>tgagttggctgctgccaccgctgagcaataactagcataaccccttggggcctctaaacgggtcttgaggggttttttgctgaaagccaatt<br>ctgattagaaaaactcatcgagcatcaaatgaaactgcaatttattcatatcaggattatcaataccatatttttgaaaaagccgtttctgtaat<br>gaaggagaaaactcaccgaggcagttccataggatggcaagatcctggtatcggtctgcgattccgactcgtccaacatcaatacaacc<br>tattaatttcccctcgtcaaaaataaggttatcaagtgagaaatcaccatgagtgacgactgaatccggtgagaatggcaaaagcttatgca<br>tttctttccagacttgttcaacaggccagccattacgctcgtcatcaaaatcactcgcatcaaccaaaccgttattcattcgtgattgcgcctg<br>agcgagacgaaatacgcgatcgctgttaaaaggacaattacaaacaggaatcgaatgcaaccggcgcaggaacactgccagcgcatc<br>aacaatattttcacctgaatcaggatattcttctaatacctggaatgctgttttcccggggatcgcagtggtgagtaaccatgcatcatcagg<br>agtacggataaaatgcttgatggtcggaagaggcataaattccgtcagccagtttagtctgaccatctcatctgtaacatcattggcaacgc<br>tacctttgccatgtttcagaaacaactctggcgcatcgggcttcccatacaatcgatagattgtcgcacctgattgcccgacattatcgcga<br>gcccatttatacccatataaatcagcatccatgttggaatttaatcgcggcttcgagcaagacgtttcccgttgaatatggctcataacaccc<br>cttgtattactgtttatgtaagcagacagttttattgttcatgatgatatatttttatcttgtgcaatgtaacatcagagattttgagacacaacgtg	
$P_{T7,strong}$ triggerA	Plasmid encoding triggerA expression from $P_{T7,strong}$
$P_{T7,strong}$ **trigger A** Terminator Kanamycin resistance cassette ColE1 origin	
agatcaaaggatcttcttgagatcctttttttctgcgcgtaatctgctgcttgcaaacaaaaaaaccaccgctaccagcggtggtttgtttgcc<br>ggatcaagagctaccaactctttttccgaaggtaactggcttcagcagagcgcagataccaaatactgttcttctagtgtagccgtagttag<br>gccaccacttcaagaactctgtagcaccgcctacatacctcgctctgctaatcctgttaccagtggctgctgccagtggcgataagtcgtg<br>tcttaccgggttggactcaagacgatagttaccggataaggcgcagcggtcgggctgaacggggggttcgtgcacacagcccagcttg<br>gagcgaacgacctacaccgaactgagatacctacagcgtgagctatgagaaagcgccacgcttcccgaagggagaaaggcggaca<br>ggtatccggtaagcggcagggtcggaacaggagagcgcacgagggagcttccagggggaaacgcctggtatctttatagtcctgtcg<br>ggtttcgccacctctgacttgagcgtcgatttttgtgatgctcgtcaggggggcggagcctatggaaaaacgccagcaacgcgatcccg	

cgaaattaatacgactcactatagggaga**GGGTCAGTTCCTGAGGTACCAGGAACTGAAACTAA**
CATCATATACACAGCCTTATTCCATCACACtagcataaccccttggggcctctaaacgggtcttgaggg
gtttttgctgaaagccaattctgattagaaaaactcatcgagcatcaaatgaaactgcaatttattcatatcaggattatcaataccatattttt
gaaaaagccgtttctgtaatgaaggagaaaactcaccgaggcagttccataggatggcaagatcctggtatcggtctgcgattccgactc
gtccaacatcaatacaacctattaatttcccctcgtcaaaaataaggttatcaagtgagaaatcaccatgagtgacgactgaatccggtgag
aatggcaaaagcttatgcatttctttccagacttgttcaacaggccagccattacgctcgtcatcaaaatcactcgcatcaaccaaaccgtta
ttcattcgtgattgcgcctgagcgagacgaaatacgcgatcgctgttaaaaggacaattacaaacaggaatcgaatgcaaccggcgcag
gaacactgccagcgcatcaacaatattttcacctgaatcaggatattcttctaatacctggaatgctgttttcccggggatcgcagtggtga
gtaaccatgcatcatcaggagtacggataaaatgcttgatggtcggaagaggcataaattccgtcagccagtttagtctgaccatctcatct
gtaacatcattggcaacgctacctttgccatgtttcagaaacaactctggcgcatcgggcttcccatacaatcgatagattgtcgcacctga
ttgcccgacattatcgcgagcccatttatacccatataaatcagcatccatgttggaatttaatcgcggcttcgagcaagacgtttcccgttg
aatatggctcataacaccccttgtattactgtttatgtaagcagacagttttattgttcatgatgatatatttttatcttgtgcaatgtaacatcaga
gattttgagacacaacgtg

$P_{T7,strong}$ triggerB	Plasmid encoding triggerB expression from $P_{T7,strong}$

$P_{T7,strong}$ **trigger B** Terminator Kanamycin resistance cassette ColE1 origin

agatcaaaggatcttcttgagatcctttttttctgcgcgtaatctgctgcttgcaaacaaaaaaaccaccgctaccagcggtggtttgtttgcc
ggatcaagagctaccaactctttttccgaaggtaactggcttcagcagagcgcagataccaaatactgttcttctagtgtagccgtagttag
gccaccacttcaagaactctgtagcaccgcctacatacctcgctctgctaatcctgttaccagtggctgctgccagtggcgataagtcgtg
tcttaccgggttggactcaagacgatagttaccggataaggcgcagcggtcgggctgaacggggggttcgtgcacacagcccagcttg
gagcgaacgacctacaccgaactgagatacctacagcgtgagctatgagaaagcgccacgcttcccgaagggagaaaggcggaca
ggtatccggtaagcggcagggtcggaacaggagagcgcacgagggagcttccagggggaaacgcctggtatctttatagtcctgtcg
ggtttcgccacctctgacttgagcgtcgatttttgtgatgctcgtcaggggggcggagcctatggaaaaacgccagcaacgcgatcccg
cgaaattaatacgactcactatagggaga**GGGATGCCCGTAGTTCATTCTACGGGCATGAATAA**
CGACATACAGCAAGCGATTTACTTATACTAtagcataaccccttggggcctctaaacgggtcttgaggg
gtttttgctgaaagccaattctgattagaaaaactcatcgagcatcaaatgaaactgcaatttattcatatcaggattatcaataccatattttt
gaaaaagccgtttctgtaatgaaggagaaaactcaccgaggcagttccataggatggcaagatcctggtatcggtctgcgattccgactc
gtccaacatcaatacaacctattaatttcccctcgtcaaaaataaggttatcaagtgagaaatcaccatgagtgacgactgaatccggtgag
aatggcaaaagcttatgcatttctttccagacttgttcaacaggccagccattacgctcgtcatcaaaatcactcgcatcaaccaaaccgtta
ttcattcgtgattgcgcctgagcgagacgaaatacgcgatcgctgttaaaaggacaattacaaacaggaatcgaatgcaaccggcgcag
gaacactgccagcgcatcaacaatattttcacctgaatcaggatattcttctaatacctggaatgctgttttcccggggatcgcagtggtga
gtaaccatgcatcatcaggagtacggataaaatgcttgatggtcggaagaggcataaattccgtcagccagtttagtctgaccatctcatct
gtaacatcattggcaacgctacctttgccatgtttcagaaacaactctggcgcatcgggcttcccatacaatcgatagattgtcgcacctga
ttgcccgacattatcgcgagcccatttatacccatataaatcagcatccatgttggaatttaatcgcggcttcgagcaagacgtttcccgttg
aatatggctcataacaccccttgtattactgtttatgtaagcagacagttttattgttcatgatgatatatttttatcttgtgcaatgtaacatcaga
gattttgagacacaacgtg

$P_{T7,strong}$ triggerH	Plasmid encoding triggerH expression from $P_{T7,strong}$

$P_{T7,strong}$ **trigger H** Terminator Kanamycin resistance cassette ColE1 origin

agatcaaaggatcttcttgagatcctttttttctgcgcgtaatctgctgcttgcaaacaaaaaaaccaccgctaccagcggtggtttgtttgcc
ggatcaagagctaccaactctttttccgaaggtaactggcttcagcagagcgcagataccaaatactgttcttctagtgtagccgtagttag
gccaccacttcaagaactctgtagcaccgcctacatacctcgctctgctaatcctgttaccagtggctgctgccagtggcgataagtcgtg
tcttaccgggttggactcaagacgatagttaccggataaggcgcagcggtcgggctgaacggggggttcgtgcacacagcccagcttg
gagcgaacgacctacaccgaactgagatacctacagcgtgagctatgagaaagcgccacgcttcccgaagggagaaaggcggaca

ggtatccggtaagcggcagggtcggaacaggagagcgcacgagggagcttccagggggaaacgcctggtatctttatagtcctgtcg
ggtttcgccacctctgacttgagcgtcgatttttgtgatgctcgtcaggggggcggagcctatggaaaaacgccagcaacgcgatcccg
cgaaattaatacgactcactatagggaga**gggaccgtggaccgcatgaggtccacggtaaacataactataacaagcctacaatt**
cattcaaactagcataaccccttggggcctctaaacgggtcttgaggggttttttgctgaaagccaattctgattagaaaaactcatcgag
catcaaatgaaactgcaatttattcatatcaggattatcaataccatatttttgaaaaagccgtttctgtaatgaaggagaaaactcaccgagg
cagttccataggatggcaagatcctggtatcggtctgcgattccgactcgtccaacatcaatacaacctattaatttcccctcgtcaaaaata
aggttatcaagtgagaaatcaccatgagtgacgactgaatccggtgagaatggcaaaagcttatgcatttctttccagacttgttcaacagg
ccagccattacgctcgtcatcaaaatcactcgcatcaaccaaaccgttattcattcgtgattgcgcctgagcgagacgaaatacgcgatcg
ctgttaaaaggacaattacaaacaggaatcgaatgcaaccggcgcaggaacactgccagcgcatcaacaatattttcacctgaatcagg
atattcttctaatacctggaatgctgttttcccggggatcgcagtggtgagtaaccatgcatcatcaggagtacggataaaatgcttgatggt
cggaagaggcataaattccgtcagccagtttagtctgaccatctcatctgtaacatcattggcaacgctacctttgccatgtttcagaaacaa
ctctggcgcatcgggcttcccatacaatcgatagattgtcgcacctgattgcccgacattatcgcgagcccatttatacccatataaatcag
catccatgttggaatttaatcgcggcttcgagcaagacgtttcccgttgaatatggctcataacacccccttgtattactgtttatgtaagcaga
cagttttattgttcatgatgatatatttttatcttgtgcaatgtaacatcagagattttgagacacaacgtg

Table S2. Concentrations of DNA or RNA templates used in all reported experiments.

Figure	Construct	Concentration
1, S2, S3, S4, S5	Empty vector	10 nM
	sfGFP plasmid	Varied 0.5-30 nM
2, S6, S7	Empty vector	10 nM
	3WJdB plasmid	Varied 0.5-30 nM, as indicated in each panel
3	mRFP plasmid	10 nM
	sfGFP plasmid	Varied 0.5-30 nM
4B	Switch B plasmid	1 nM
	Trigger A plasmid	30 nM
	Trigger B plasmid	30 nM
	Trigger H plasmid	30 nM
4C	Switch B plasmid	1 nM
	Trigger A RNA	2 µM
	Trigger B RNA	2 µM
	Trigger H RNA	2 µM
5B	LacI-sfGFP plasmid	Varied 0.01-10 nM
	mRFP plasmid	10 nM
5C	LacI-sfGFP plasmid	10 nM
	mRFP plasmid	10 nM
S1	All plasmids	5 nM
S8	LacI-sfGFP plasmid	Varied 0.01-10 nM
	mRFP plasmid	10 nM
S10	sfGFP plasmid	Varied 0.5-2.5 nM
	EmptySigma70	10 nM

Table S3. Initial parameter values and search space lower and upper boundaries for the simulation results in Figure S11. Initial guesses were sourced from Singhal *et al., Synthetic Biology*, 2021. The lower and upper boundaries indicate the boundary for the parameter search space. Some parameters were kept constant in all runs.

Parameter	Initial value	Lower boundary	Upper boundary
TX_elong_glob_T7	12.418	1.2418	124.18
TX_transcription_t7utrbroc_no_protein	0.081697368	0.008169737	0.816973684
TX_NTPcons_t7utrbroc_no_protein	3.022802632	0.302280263	30.22802632
TX_elong_glob	12.418	1.2418	124.18
TX_transcription_utrbroc_no_protein	0.081697368	0.008169737	0.816973684
TX_NTPcons_utrbroc_no_protein	3.022802632	0.302280263	30.22802632
TX_transcription_utrkanR_kanR	0.015218137	0.001521814	0.152181373
TX_NTPcons_utrkanR_kanR	3.089281863	0.308928186	30.89281863
TL_elong_glob	23.826	2.3826	238.26
TL_translation_kanR	0	0	0
TL_REScons_kanR	0	0	0
TX_transcription_t7utrempty_no_protein	0.243490196	0.02434902	2.434901961
TX_NTPcons_t7utrempty_no_protein	2.921882353	0.292188235	29.21882353
TX_transcription_utrempty_no_protein	0.243490196	0.02434902	2.434901961
TX_NTPcons_utrempty_no_protein	2.921882353	0.292188235	29.21882353
AGTPreg_varying	0.02	0.002	0.2
AGTPdeg_time	6529.5	652.95	65295
AGTPreg_ON	0.02	0.002	0.2
AGTPdeg_rate	0.0000487	0.00000487	0.000487
TXTL_PT7_RNAPbound_F	0.01	0.01	0.01
TXTL_PT7_RNAPbound_R	0.1	0.01	1
TXTL_RNAPBOUND_TERMINATION_RATE	1.7912	0.17912	17.912
TXTL_NTP_RNAP_1_F	1	1	1
TXTL_NTP_RNAP_1_R	19.028	1.9028	190.28
TXTL_NTP_RNAP_2_F	1	1	1
TXTL_NTP_RNAP_2_R	1199001.878	119900.1878	11990018.78
TXTL_RNAdeg_F	1	1	1
TXTL_RNAdeg_R	3690000	369000	36900000
TXTL_RNAdeg_kc	0.087664	0.0087664	0.87664
TXTL_PT773_RNAPbound_F	0.01	0.01	0.01
TXTL_PT773_RNAPbound_R	0.1	0.01	1
TXTL_PJ23119_RNAPbound_F	0.01	0.01	0.01
TXTL_PJ23119_RNAPbound_R	1	0.1	10
TXTL_UTR_kanR_F	0.81873	0.81873	0.81873
TXTL_UTR_kanR_R	59219	5921.9	592190

TXTL_PkanR_RNAPbound_F	0.01	0.01	0.01
TXTL_PkanR_RNAPbound_R	0.1	0.01	1
TL_AA_F	0.7408	0.7408	0.7408
TL_AA_R	521.443	52.1443	5214.43
TL_AGTP_F	0.3012	0.3012	0.3012
TL_AGTP_R	602590	60259	6025900
TXTL_RIBOBOUND_TERMINATION_RATE	12.984	1.2984	129.84
RNAP_0	4.7367	0.47367	47.367
t7RNAP_0	4.7367	0.47367	47.367
RNase_0	30269	3026.9	302690
Ribo_0	266.42	26.642	2664.2
